# A Metabolome Analysis and the Immunity of *Phlomis purpurea* against *Phytophthora cinnamomi*

**DOI:** 10.3390/plants12101929

**Published:** 2023-05-09

**Authors:** Dina Neves, Andreia Figueiredo, Marisa Maia, Endre Laczko, Maria Salomé Pais, Alfredo Cravador

**Affiliations:** 1 Faculdade de Ciências e Tecnologia, Universidade do Algarve, Campus de Gambelas, 8005-139 Faro, Portugal; 2 Grapevine Pathogen Systems Lab (GPS Lab), Biosystems & Integrative Sciences Institute (BioISI), Faculdade de Ciências da Universidade de Lisboa, Campo Grande, 1749-016 Lisboa, Portugal; 3 Departamento de Biologia Vegetal, Faculdade de Ciências da Universidade de Lisboa, Campo Grande 016, 1749-016 Lisboa, Portugal; 4 Functional Genomics Center, UZH/ETHZ, Winterthurerstr. 190, CH-8057 Zürich, Switzerland; 5 Academia das Ciências de Lisboa, R. da Academia das Ciências de Lisboa, 19, 1200-168 Lisboa, Portugal; 6 MED—Mediterranean Institute for Agriculture, Environment and Development & CHANGE—Global Change and Sustainability Institute, Universidade do Algarve, Campus de Gambelas, 8005-139 Faro, Portugal

**Keywords:** *Phlomis purpurea*, *Phytophthora cinnamomi*, biotic stress, metabolomic and transcriptomic integration, plant immune response

## Abstract

*Phlomis purpurea* grows spontaneously in the southern Iberian Peninsula, namely in cork oak (*Quercus suber*) forests. In a previous transcriptome analysis, we reported on its immunity against *Phytophthora cinnamomi*. However, little is known about the involvement of secondary metabolites in the *P. purpurea* defense response. It is known, though, that root exudates are toxic to this pathogen. To understand the involvement of secondary metabolites in the defense of *P. purpurea,* a metabolome analysis was performed using the leaves and roots of plants challenged with the pathogen for over 72 h. The putatively identified compounds were constitutively produced. Alkaloids, fatty acids, flavonoids, glucosinolates, polyketides, prenol lipids, phenylpropanoids, sterols, and terpenoids were differentially produced in these leaves and roots along the experiment timescale. It must be emphasized that the constitutive production of taurine in leaves and its increase soon after challenging suggests its role in *P. purpurea* immunity against the stress imposed by the oomycete. The rapid increase in secondary metabolite production by this plant species accounts for a concerted action of multiple compounds and genes on the innate protection of *Phlomis purpurea* against *Phytophthora cinnamomi*. The combination of the metabolome with the transcriptome data previously disclosed confirms the mentioned innate immunity of this plant against a devastating pathogen. It suggests its potential as an antagonist in phytopathogens’ biological control. Its application in green forestry/agriculture is therefore possible.

## 1. Introduction

Via natural selection, plants evolved the capacity to survive and adapt to changing environments by producing a vast array of secondary metabolites. Secondary metabolites usually function in plants as a chemical barrier raised against a pathogen attack and invasion as part of their defense strategy [1]. They constitute an unusually large group of structurally diversified compounds that can be produced in plants by various primary metabolites or their biosynthetic intermediates, constitutively or in response to different environmental stimuli [2]. Biosynthesis and the activation of secondary metabolites are triggered by the recognition of microbe-associated molecular patterns (MAMPs) by pattern recognition receptors or the detection of microbial effectors by resistance proteins [3].

A large array of secondary metabolites are involved in plant immunity and may vary among plant species. A frequently used classification is based on the mode of biosynthesis and the accumulation of defense-related phytochemicals. They are generally categorized according to the timing of their synthesis: (1) phytoanticipins, if they are produced before a challenge with microorganisms or produced after an infection solely from preexisting precursors, and (2) phytoalexins, if they are de novo synthesized and rapidly accumulated upon a pathogen infection [4,5,6,7,8]. There are several chemical classes and structural types of secondary metabolites, such as phenolic compounds, isoprenoids or terpenoids, alkaloids, fatty acid derivatives and polyketides, shikimates, benzoxazinoids, glucosinolates and their metabolic products, saponins, cyanogenic glycosides, and organosulfur compounds [1,9].

Among the Lamiaceae, the *Phlomis* species have attracted the most attention across Asia and the Mediterranean basin, not only to identify their secondary metabolites, but also to look for new substances with potential therapeutic effects [10]. A profusion of secondary metabolites from these species has been isolated and identified, some of them presenting pharmacological activity [11,12,13,14,15]. Antioxidant, cytotoxic, anti-inflammatory, and antimicrobial (antibacterial, antifungal, and antiviral) are examples of biological activities with potential benefits for human health [16,17,18]. Ramos da Silva et al. [19]) and Correia et al. [20] have emphasized the properties of *Lamiaceae* essential oils as antioxidants. Other biological activities have been reported, such as the antifungal properties used in food preservation [21].

Neves et al. [22,23] demonstrated that *Phlomis purpurea* L. (purple phlomis), a perennial species of the Lamiaceae family, growing in the vicinity of cork oak (*Quercus suber*) and holm oak (*Quercus ilex* ssp *rotundifolia*) confers protection against the widely spread and highly aggressive *Phytophthora cinnamomi* phytopathogen, a causal agent of devastating diseases in thousands of hosts, including cork and holm oaks. This protective effect may be related to the exudation to the rhizosphere of the triterpenoid, phlomispurpentaolone, which has been demonstrated to be toxic to the *Phytophthora* species [24].

In order to understand the mechanisms underlying the ability of *Phlomis purpurea* to reduce disease spread, a transcriptome analysis of *Phlomis purpurea* challenged with *Phytophthora cinnamomi* has been produced and a set of genes and hormones acting in a concerted manner has been identified, accounting for both elicited and constitutive defense [25,26]. Among them, several transcriptional factors shown to regulate the secondary metabolite biosynthesis in plants under stress conditions have been identified by other authors [25,26]. In addition, terpenoids and polyketides, as well as other secondary metabolites, lipids, and carbohydrate metabolism were among the top six pathways more represented by the unigenes annotated [25]. Altogether, these results raised the expectations of discovering the putative metabolites overproduced as a response to the stress imposed by a pathogen.

The receptors involved in pathogen recognition and the resistance proteins detecting the pathogen effectors, as well as the defense-related genes transcriptionally activated by downstream signaling, have been disclosed [25,26,27]. However, the contribution of secondary metabolites to plant immunity has been less studied.

Here, we present the results of a metabolome analysis on the leaves and roots of *Phlomis purpurea* challenged with *Phytophthora cinnamomi* and discuss the role of putative metabolites and transcripts on the innate defense against the pathogen *Phytophthora cinnamomi*, as a contribution to understanding the mechanisms underlying *Phlomis purpurea* immunity.

## 2. Results

### 2.1. Phlomis purpurea Metabolome Profiling after Challenge with Phytophthora cinnamomi

A *Phlomis purpurea* metabolome modulation with *Phytophthora cinnamomi* was analyzed in both its leaves and root tissues using an untargeted LC-MS/MS approach. The roots from whole seedlings were used for a challenge with zoospores. The samples were collected at 6, 12, 24, 48, and 72 h post root challenge (hpc). The controls were collected at the same time points. Ten replicates were prepared for each time point and the experience was repeated five times. The mass spectra of the leaves or roots from the challenged and non-challenged *P. purpurea* were compared and the results, presented as supplementary data, indicate considerable differences in the metabolite composition at all the time points in the leaves and roots, as illustrated in Supplementary Figure S1.

From the initial 6989 ion peaks detected in the positive ionization mode in the leaves, 1566 putative metabolites belonging to various chemical groups were putatively identified (Supplementary Table S1). Among those, 134 differentially accumulated *m*/*z* were identified and up to 42% were considered as putative metabolites (Table 1). In total, 6503 ion peaks were detected in the roots and 1316 putative metabolites belonging to various chemical groups were putatively identified (Supplementary Table S2). A total of 147 *m*/*z* were differentially accumulated along the time points analyzed and only 23% were identified as putative metabolites (Table 1). At 24 hpc, although ten ions presented a differential accumulation between the challenged and non-challenged samples, no correspondence of the *m*/*z* values to known metabolites was found in the database search.

### 2.2. Leaf Metabolome Modulation

**Figure 1:**
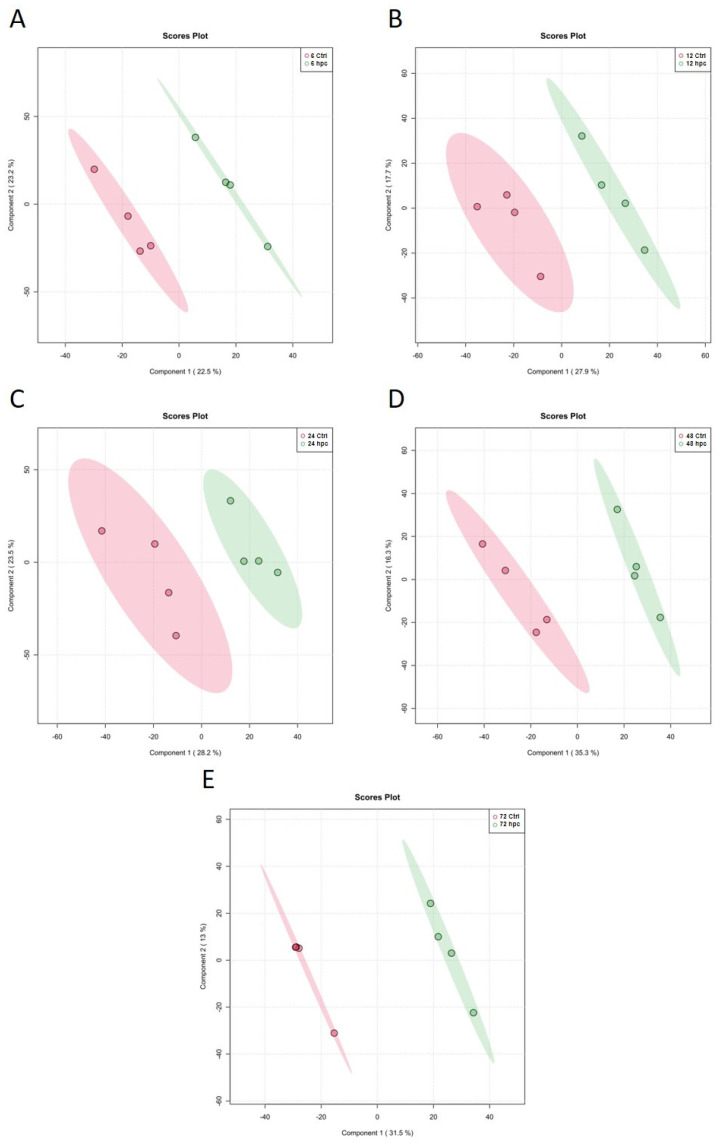
Partial least squares discriminant analysis (PLS-DA) components 1 and 2 score plots of *Phlomis purpurea* leaf metabolite profiles at (**A**) 6, (**B**) 12, (**C**) 24, (**D**) 48, and (**E**) 72 hpc with *Phytophthora cinnamomi*. In the score plots, the ellipse represents the Hotelling T2 with 95% confidence interval. The red ellipses represent the controls and the green ellipses the challenged samples. Four biological replicates were performed per analysis.

A data matrix considering the ions detected at all the time points was submitted to the MetaboAnalyst R package for statistical analysis. A supervised statistical approach, a partial least squares discriminant analysis (PLS-DA), was applied to the untargeted metabolomics data. The multivariate analysis revealed a separation between the challenged and control samples at all the studied time points (Figure 1), which suggests that a *Phytophthora cinnamomi* attack influences the *Phlomis purpurea* leaf metabolic profile, enabling discrimination between the control and challenged samples. The combined analysis of both components (C1 and C2) for all the different time points accounts for over 45% of the sample variation. Both the components account for variations of 45.7%, 45.6%, 51.7%, 51.6%, and 44.5% for 6, 12, 24, 48, and 72 hpc, respectively. To understand the biological relevance of these differences, the *m*/*z* values were putatively annotated based on the KEGG and LipidMaps databases (Supplementary Table S3). Bibliographic searches and the KNApSAcK database were used to confirm that the putatively identified metabolites were assigned to the plant kingdom. A total of 56 *m*/*z* values were putatively annotated and presented a significant modulation (*p* ≤ 0.05).

**Figure 2:**
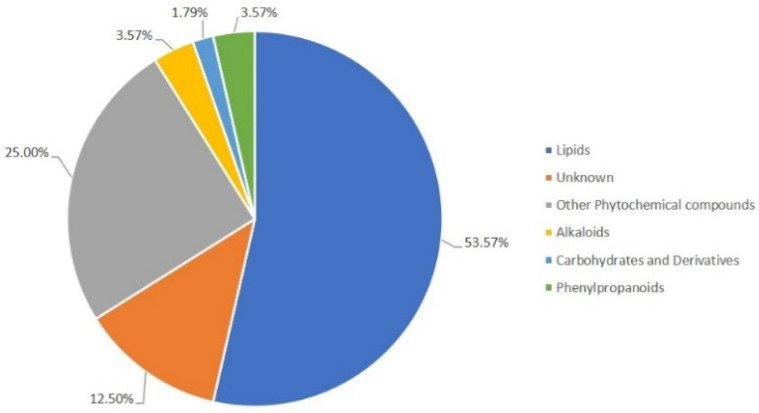
Metabolic secondary classes’ annotation of *Phlomis purpurea* leaf for the 6, 12, 24, 48, and 72 hpc with *Phytophthora cinnamomi* according to the KEGG and LipidMaps classification.

The predicted metabolites from the *m*/*z* peaks, i.e., the observed ions, were putatively assigned to different metabolic classes, with the most predominant being lipids (53.57%), phytochemical compounds (25%), alkaloids (3.57%), phenylpropanoids (3.57%), and carbohydrates (1.79%) (Figure 2). Some metabolites (12.50%) were putatively assigned, although their function is not yet known. A detailed list of the putative metabolites for each of the time points analyzed is presented in Supplementary Table S3.

**Figure 3:**
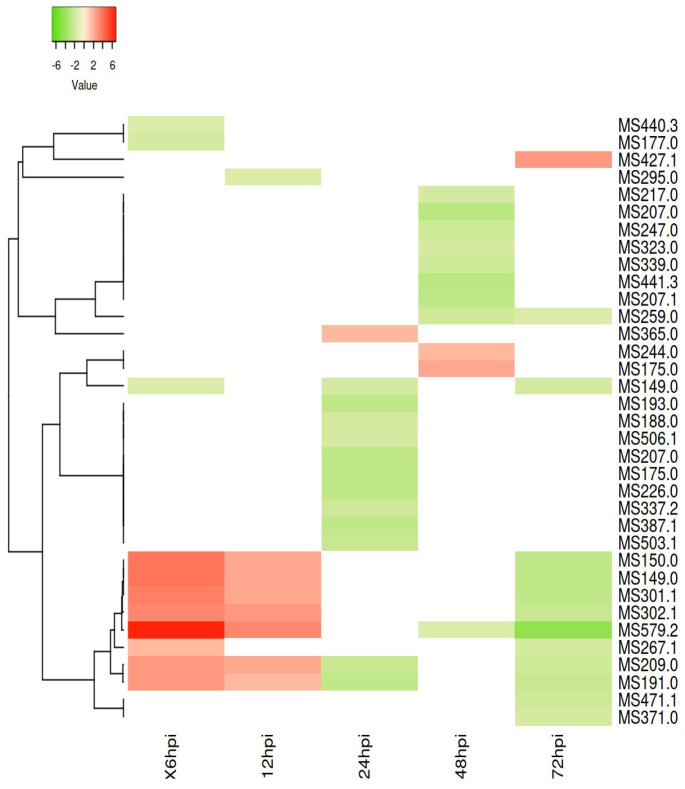
Heatmap of differently accumulated ions in each metabolic class from leaves of *Phlomis purpurea* challenged at 6, 12, 24, 48, and 72 hpc with *Phytophthora cinnamomi*. Each column indicates the time point (6, 12, 24, 48, and 72 hpc). Each row represents a feature (*m*/*z* value) putatively identified as a metabolite in leaves of *Phlomis purpurea* challenged with *Phytophthora cinnamomi*. Coloring indicates differential accumulation between challenged and control samples: green indicates low accumulation (fold change < 1), light red indicates significant accumulation (fold change between 2 and 4), and red indicates high accumulation (fold change > 6).

To further evaluate the modulation of the different metabolic classes, a heatmap using all the *m*/*z* values, i.e., the observed ions, putatively annotated from the different time points, was constructed. After 12 h of the *Phlomis purpurea* being challenged with the *Phytophthora cinnamomi,* a positive modulation of the ions with *m*/*z* values of 579.26, 149.0069, 150.0082, 301.126, 302.13, 191.052, 209.066, and 267.155, mostly associated with lipids and phytochemical compound classes, was observed in the leaves compared to the control samples (Figure 3). From 24 hpc to 72 hpc, an overall decrease in the same masses occurred. In the challenged samples, most of the differentially accumulated metabolites presented a negative modulation at 24, 48, and 72 hpc. A metabolite putatively assigned as an alkaloid was up-accumulated at 24 hpc (*m*/*z* value 365.0), two (*m*/*z* values 175.0 and 244.0) were assigned as lipids. From the metabolites up-accumulated at 48 hpc 12.5% could not be identified. A putative metabolite (*m*/*z* value 427.1) assigned as a phytochemical compound was up-accumulated at 72 hpc.

### 2.3. Root Metabolome Modulation

**Figure 4:**
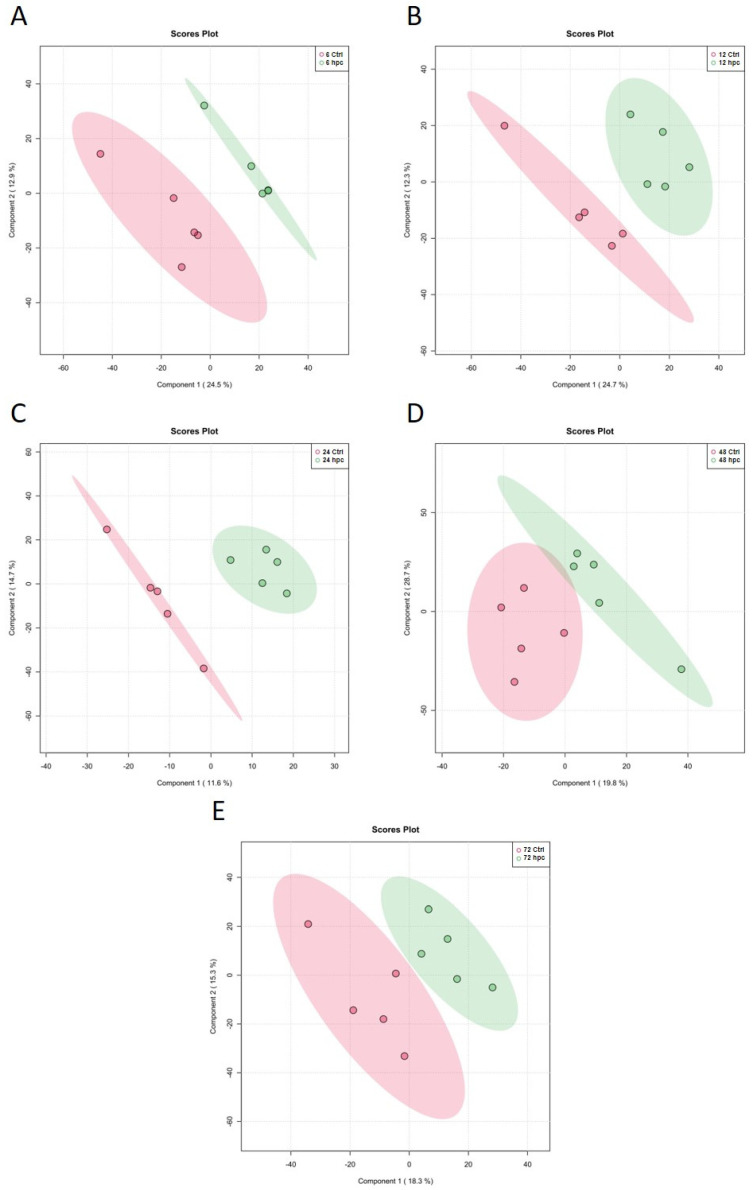
Partial least squares discriminant analysis (PLS-DA) components 1 and 2 score plots of *Phlomis purpurea* roots metabolite profiles at (**A**) 6, (**B**) 12, (**C**) 24, (**D**) 48, and (**E**) 72 hpc with *Phytophthora cinnamomi*. In the score plots, the ellipse represents the Hotelling T2 with 95% confidence interval. The red ellipses represent the controls and the green ellipses the challenged samples. Five biological replicates were performed per analysis.

The PLS-DA analysis of the *Phlomis purpurea* roots challenged with *Phytophthora cinnamomi* revealed a separation between the challenged and control samples for all the time points studied (Figure 4), representing a response modulation of the metabolism upon a pathogen attack. In the roots, the separation was not as evident as that in the leaf samples. For the different time points, components 1 and 2 accounted for over 30% of the sample variation (37.4%, 37%, and 33.6% for 6, 12, and 72 hpc, respectively), except for 24 hpc, which had a global explanation of 26.3%. The maximum separation obtained for both components was at 48 hpc with 48.5%. For both the leaf and root tissues, 48 hpc was the time point with the highest metabolic modulation between the control and challenged samples.

**Figure 5:**
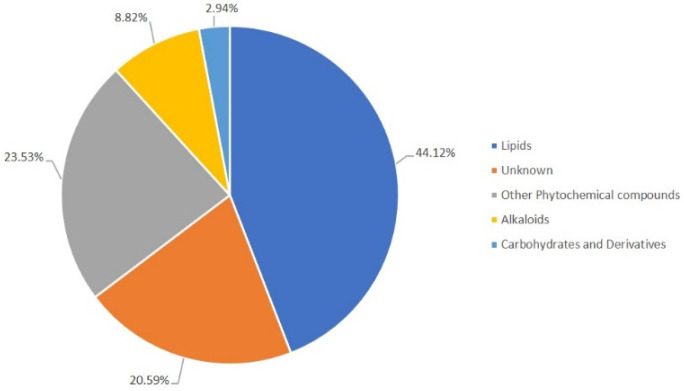
Metabolic secondary classes’ annotation of *Phlomis purpurea* roots for the 6, 12, 24, 48, and 72 hpc with *Phytophthora cinnamomi* according to the KEGG and LipidMaps classification.

In the roots, a total of 34 ions putatively annotated presented significant modulation (*p* ≤ 0.05) between the challenged and control samples. The predicted root-associated metabolites were from the lipids (44.12%), phytochemical compounds (23.53%), alkaloids (8.82%), and carbohydrates (2.94%) classes (Figure 5). A percentage (20.59%) of the putative metabolites were not assigned to any functional class but were found to occur in the plant kingdom. Complete lists of the putative metabolites for each of the time points analyzed are given in Supplementary Table S4.

**Figure 6:**
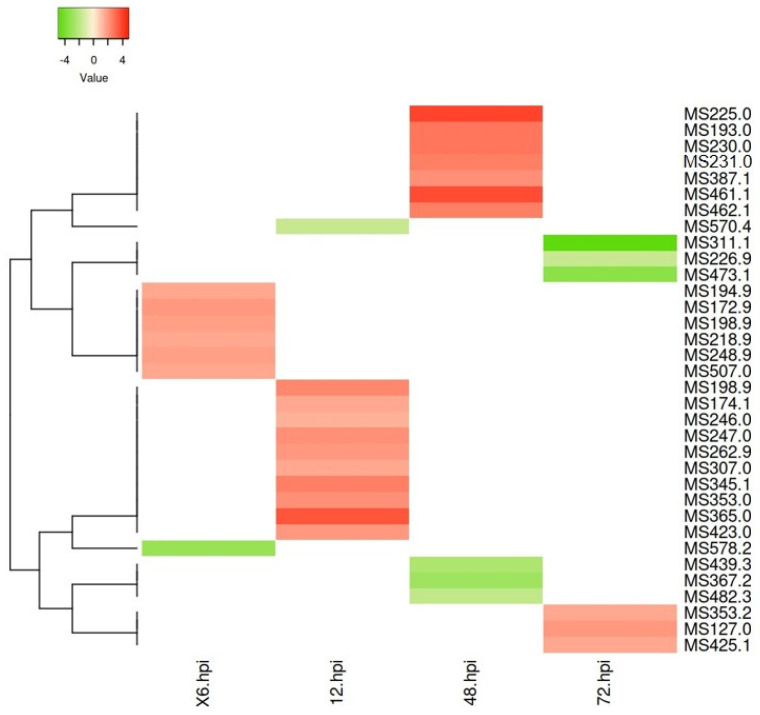
Heatmap of differently accumulated ions in each metabolic class from *Phlomis purpurea* roots challenged at 6, 12, 48, and 72 hpc with *Phytophthora cinnamomi* (no metabolites were assigned for the 24 hpc). Each column indicates the time point (6, 12, 48, and 72 hpc). Each row represents a feature, i.e., ion putatively identified as a metabolite in *Phlomis purpurea* roots challenged with *Phytophthora cinnamomi*. Coloring indicates differential accumulation between challenged and control samples: green indicates low accumulation (fold change < 1), light red indicates significant accumulation (fold change between 2 and 3), and red indicates high accumulation (fold change > 4).

After the first 48 h of interaction, a positive modulation of most metabolites was observed in the challenged samples compared to the controls (Figure 6).

At the first hours of interaction (6 and 12 hpc), this modulation was associated with alkaloids (e.g., *m*/*z* 262.9, *m*/*z* 174.1, and *m*/*z* 246.0) and other phytochemical compounds (e.g., *m*/*z* 365.0, *m*/*z* 345.1, *m*/*z* 353.0, and *m*/*z* 423.0). Other ions presented a positive modulation but no annotation was available in the databases. At 48 and 72 h, the number of positively modulated putatively identified metabolites was reduced in the challenged samples compared to the controls. For these time points, some ions (*m*/*z* 461.1, *m*/*z* 230.0, *m*/*z* 231.0, *m*/*z* 387.1, *m*/*z* 127.0, and *m*/*z* 353.2) were putatively annotated as lipids and others (*m*/*z* 225.0, *m*/*z* 193.0, *m*/*z* 462.1, and *m*/*z* 425.1) as phytochemical compounds.

### 2.4. Metabolite Class Analysis

**Figure 7:**
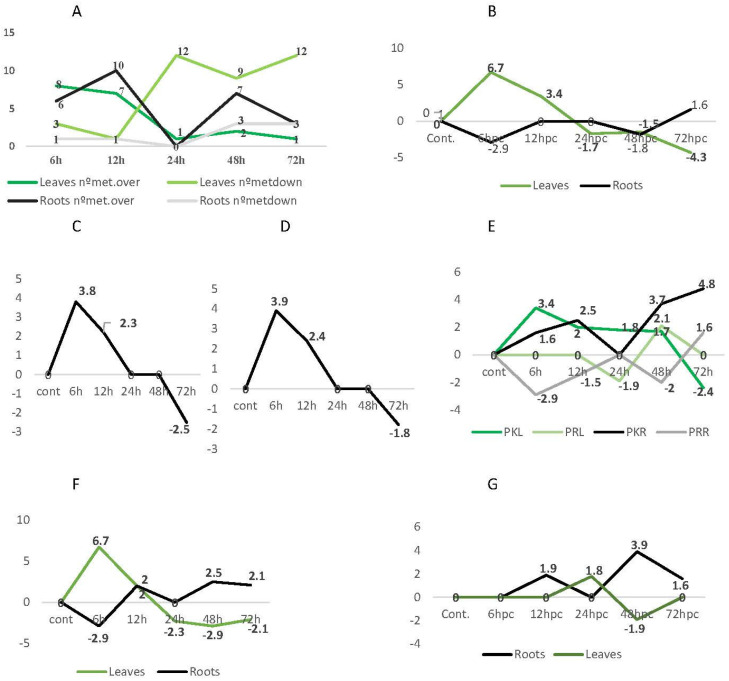

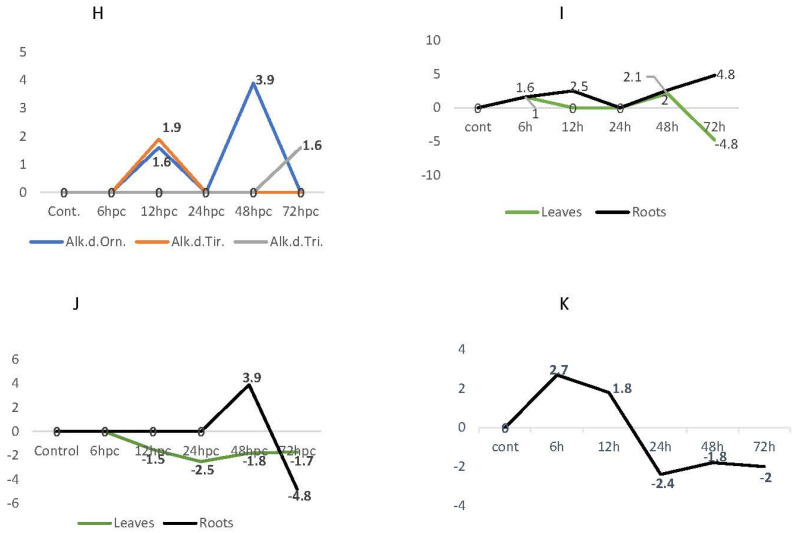
Number of putative metabolites of *Phlomis purpurea* over- and down-produced in leaves and roots (**A**) and their fold change profiles along the time schedule after challenging with *Phytophthora cinnamomi.* Terpenoids (**B**); taurine (**C**); and imidazole-4-acetaldehyde (**D**) in leaves; polyketides (PK) and prenol lipids (PR) in leaves (L) and roots (R) (**E**); fatty acyls (**F**); alkaloids (**G**); sub-classes of alkaloids in roots (**H**); flavonoids (**I**); phenylpropanoids (**J**); and vitamins and other phytochemicals in leaves (**K**).

The metabolome analysis of the leaves and roots, performed along the challenging time, allowed for the identification of the putative metabolites up- or down-accumulated by *Phlomis purpurea* after contact with the pathogenic oomycete *Phytophthora cinnamomi*. Our results revealed that, in the leaves, the number of over-produced metabolites decreased from 8 to 1 between 6 and 72 hpc. On the contrary, the down-produced metabolites increased along the challenge time course (Figure 7A). In the roots, the number of over-produced metabolites increased from 6 to 10 between 6 and 12 hpc, decreasing thereafter to 3 at 72 hpc. The number of down-produced metabolites increased from 1 to 3 along the challenge time course. Although the roots were challenged by the pathogen, the leaves underwent the greatest metabolic change.

#### 2.4.1. Terpenoids

The comparison of the terpenoids fold change profiles in the leaves with those from roots revealed a very quick response in the leaves at (6.7 at 6 hpc), decreasing to negative values (−4.3) at 72 hpc. An opposite profile occurred in the roots (Figure 7B).

#### 2.4.2. Taurine and Imidazole-Acetaldehyde

In the challenged leaves, taurine accumulated over a 3.9 fold change at 6 hpc, decreasing at 12 hpc, and attaining values of −1.8 at 72 hpc (Figure 7C). Imidazole-4-acetaldehyde followed a similar tendency, with a 3.8 fold change at 6 hpc and −2.5 at 72 hpc (Figure 7D). In the roots, these two compounds were not identified.

#### 2.4.3. Polyketides, Prenol Lipids and Fatty Acyls

In the leaves, polyketides (PKL) presented a rapidly increased production at 6 hpc (3.4 fold), decreasing to 1.7 fold at 48 hp. A negative value (−2.4 fold) was observed at 72 hpc (Figure 7E). In the roots, polyketides (PKR) were over-produced at 6, 12, 48, and 72 hpc, with fold change values of 1.6, 2.5, 3.7, and 4.8, respectively.

In the leaves, prenol lipids (PRL) decreased to negative values (−1.9 fold) at 24 hpc, increasing immediately to 2.1 fold at 48 hpc, and decreasing again to 0 at 72 hpc. In the roots, prenol lipids (PRR) were down-produced, with fold change values of −2.9 and −2 at 6 and 48 hpc, respectively, increasing at 72 hpc to a positive value of 1.6 fold (Figure 7E).

In the leaves, fatty acyls had a fold change maximal value of 6.7 at 6 hpc, decreasing to negative values from 24 to 72 hpc (Figure 7F). An inverse profile was observed for fatty acyls in the roots, with negative values (−2.9) at 6 hpc, increasing thereafter at 12 hpc (2), 48 (2.3), and 72 (2.1) hpc (Figure 7F).

#### 2.4.4. Alkaloids

In the leaves, alkaloids presented a maximal fold change of 1.8 at 24 hpc, decreasing to −1.9 at 48 hpc (Figure 7G). In the roots, alkaloids presented positive or zero-fold change values, with 1.9 at 12 hpc, decreasing at 24 hpc, and increasing again to 3.9 at 48 hpc, decreasing thereafter to 1.6 at 72 hpc (Figure 7G).

As for the other classes of compounds, the alkaloids production profile was inverse in the leaves and roots, attaining in the roots a maximum value (3.9 fold change) at 48 hpc and in the leaves, a minimum (−1.9 fold change) at the same timepoint.

In the roots, the sub-classes of alkaloids presented different profiles. In the alkaloids derived from ornithine, the fold changes were positive at 12 hpc (1.6 fold) and 48 hpc (3.9 fold), decreasing thereafter. The alkaloids derived from tyrosine increased from 6 (1 fold) to 12 hpc (3 fold), decreasing thereafter. The alkaloids derived from tryptophane had a positive accumulation (1.6 fold) only at 72 hpc (Figure 7H).

#### 2.4.5. Flavonoids

In the leaves, flavonoids showed a 1.6 fold change at 6 hpc, increasing to 2.1 at 48 hpc, and decreasing to −4.8 at 72 hpc (Figure 7I). In the roots, flavonoids showed a fold change of 1.6 at 6 hpc, which increased to 2.6 and 4.8 at 48 hpc and 72 hpc, respectively. When comparing the flavonoids in the leaves and roots along the challenging time, an inverse profile was observed from 12 to 72 hpc (Figure 7I).

#### 2.4.6. Phenylpropanoids

In the leaves, negative fold changes were observed for phenylpropanoids from 12 hpc until 72 hpc. In the roots, phenylpropanoids presented a maximum fold change of 3.9 at 48 hpc, decreasing to negative values (−4.8) at 72 hpc (Figure 7J).

#### 2.4.7. Other Compounds

Vitamins and other phytochemical compounds presented a content evolution that was higher at 6 hpc (2.7 fold), decreasing at 24, 48, and 72 hpc when compared to the controls (Figure 7K). Sterols were down-accumulated in the roots at both 12 and 48 hpc (−1.6 and −1.9 fold, respectively). Glucosinolates appeared to be accumulated only in the leaves at 72 hpc with a 2.8 fold change when compared to the control samples.

## 3. Discussion

The success of plants’ survival is significantly based on their ability to rapidly recognize specific environmental signals and biotic attacks and promote signal transduction pathways that lead to the biosynthesis of defensive compounds [28]. Immunity and agents that increase immunity have long been investigated in humans. In plants, only recently has intensive research been performed to understand the factors controlling plant immunity, especially with regard to their defense against biotic stress. In a previous work, based on data from the transcriptome analysis, we suggested that *Phlomis purpurea* presents an innate immunity against the oomycete *Phytophthora cinnamomi* [26]. Combining the transcriptome data with the metabolome results, here, we discuss the interplay of multi-compound metabolite classes and the corresponding genes coding for the enzymes participating in different metabolic pathways, reinforcing the assumption that *Phlomis purpurea* present an innate immunity against the oomycete *Phytophthora cinnamomi.* The constitutive expression and rapidly increased production of imidazole-4-acetaldehyde and taurine must be emphasized, as these compounds are very well known as being responsive for human immunity. To the best of our knowledge, this is the second paper reporting on taurine production by angiosperms infected with a pathogen and the first associating this compound and imidazole-4-acetaldehyde with plants’ innate immunity.

In combining the transcriptome results with the metabolome ones presented here and comparing these findings with the available literature, we find support for considering that *Phlomis purpurea* is a good example of plant innate immunity against the oomycete *Phytophthora cinnamomi*, opening avenues for a better understanding of plants’ immunity against plant pathogens.

A major mechanism by which plants interact and adapt to their environments is through the production of specialized metabolites [29]. Novel tools such as metabolome and transcriptome analyses deepen our knowledge of plants’ defenses against biotic and abiotic stresses. Metabolomics is a powerful tool for disclosing the molecules that are involved in the response of plants to adverse environmental conditions. The integration of metabolome with transcriptome data is crucial for understanding plants’ molecular mechanisms underlying the *Phlomis purpurea* immunity against *Phytophthora cinnamomi.* Transcription factors such as WRKY, MYB, AP2, ERF, bZIP, bHLH, CYP, and NAC, known to be involved in the biosynthesis of secondary metabolites [30,31], were up-regulated in *Phlomis purpurea* upon a challenge with *Phytophthora cinnamomi*, and their role in the innate immunity of *Phlomis purpurea* has been previously reported [25,26]. A metabolome screening of *Phlomis purpurea* leaves and roots challenged with *Phytophthora cinnamomi* revealed a set of secondary metabolites that were differentially produced along the challenge time course. Despite the plants being challenged by the pathogen in their roots, it was in the leaves that the greatest metabolic changes occurred. These results suggest that *Phlomis purpurea* quickly perceive long-distance signals in their leaves, where secondary metabolites, in particular those depending on chloplastidial metabolism, increase their production. This assumption finds support from the plastidial localization of GPP synthase (GPPS), the enzyme responsible for the synthesis of GPP as a precursor for vital metabolic branches, including specialized metabolites. The participation of chloroplasts in the biosynthesis of a large number of biomolecules (fatty acids, amino acids, sugars, vitamins, terpenes, alkaloids, and phenols, among others) is well documented [32]. The compartmentation of some terpenes and terpenoids in chloroplasts is also known. Some of the classes of compounds identified are discussed, correlating the metabolome with the transcriptome data previously associated with the innate immunity of *Phlomis purpurea* against *Phytophthora cinnamomi*.

### 3.1. Terpenoids

Terpenoids represent the largest class of chemicals among those produced by plants [33]. The role of terpenoids in plant resistance to fungal pathogens has long been investigated. They play a critical role in the defense of plants against biotic stress [34,35]. Mixtures of mono- and sesquiterpenes are associated with an increased resistance against fungal pathogens [36]. *Phlomis purpurea* responds very quickly (6 hpc) to the presence of *Phytophthora cinnamomi* by increasing the constitutive production of terpenoids in its leaves, which decreases after 24 hpc. According to Loreto et al. [37], emissions of biogenic volatile organic compounds (BVOCs) occur at the leaf, root, and flower levels and may be a constitutive characteristic or induced by a combination of biotic and abiotic stresses. Mateus et al. [24] have identified a nortriterpenoid (phlomispurpentaolone) exudated by the roots that inhibits *P. cinnamomi* growth. Nonvolatile terpenoids can be exuded from the roots into the rhizosphere and the surrounding soil environment, where they are involved in different defense responses [38], which is in accordance with our data on *P. purpurea*. The comparative transcriptome analysis of the rice variety Digu, resistant to *Magnaporthe oryzae,* revealed that the biosynthesis of terpenes is activated very soon after a challenge with the pathogen [39]. The role of terpenoids in the resistance of *Vitis vinifera* against downy mildew has also been demonstrated [40]. The transcriptome analysis of *Phlomis purpurea* challenged with *Phytophthora cinnamomi* revealed the up- regulation of several transcripts related to the production of terpenoids, namely the monoterpenes, limonene, terpineol, myrcene, geraniol, and linalool, as well as of sesquiterpenes and their precursor germacrene. Their over-expression value decreased from 24 hpc [25] in a similar way to the metabolome profile of the terpenoids in leaves, opposite to the terpenoids profile in the roots. These data suggest their translocation from the leaves to the roots, from where they are released into the soil infested by *P. cinnamomi,* inhibiting its growth. In parallel, the transcriptome of *Phlomis purpurea* challenged with *Phytophthora cinnamomi* revealed a high up-regulation of jasmonate (JA) [25]. Wei et al. [41] reported on the role of JA in terpenes biosynthesis. Zhang et al. [42] correlated its increased production with strawberry resistance to *Botrytis cinerea* infection. Regarding salicylate (SA), the transcriptome analysis revealed its high up-regulation at 12 hpc, which decreased thereafter [26]. This up-regulation may also be correlated with the metabolite profile of terpenoids in the leaves. In *Salvia macrosiphon* under stress conditions, SA may change the secondary metabolites and their pathways through changes in the plastid’s chlorophyll level [43]. Sadeghian et al. [44] reported on the enhanced production of primary and secondary metabolites after the application of SA to the Lamiaceae *Salvia khuzistanica*. Liu et al. [45] pointed out the potential role of methyl salicylate (MeSA) in plant stress signal transduction through influencing the secondary metabolism and signaling pathways, including the shikimate/phenylpropanoid (PAL) pathway. According to the same authors, MeSA elicits emissions of benzenoids (BZ), monoterpenes (MT), and fatty-acid-derived compounds (LOX products). Luo et al. (2019), through a transcriptome integrated analysis, reported on the coordinate defense response of poplar upon a fungal infection. These authors uncovered the possible roles of JA/SA in regulating the balance between growth and defense responses by integrating multiple hormone signaling pathways. They pointed out that the highest number of representative transcription factors include ERF, MYB/MYB-related genes, and bHLH. This is in agreement with our previous report on a transcriptome analysis [25,26], which revealed a high expression of ERF, MYB/MYB-related genes, and bHLH, among others.

Defense against biotrophic pathogens has been generally considered to rely on SA-associated responses, whereas JA is required for an effective response towards necrotrophic pathogens. *Phytophthora cinnamomi* notably induces both the JA and SA pathways in *Phlomis purpurea. Phytophthora cinnamomi* has been described both as a necrotroph and a hemi-biotroph [46,47,48], which could account for the involvement of both JA and SA in the response of the plant against this pathogen.

Upon combining the transcriptome analysis reported before with the metabolome analysis, we can suggest that the activation of both the JA and SA biosynthetic pathways and the terpenoid accumulation modulation might be responsible for the phytohormone-mediated innate immunity of *Phlomis purpurea* against *Phytophthora cinnamomi*.

### 3.2. Lipids

Based on their solubility and polarity, lipids are divided into eight major categories [49]. Among these are fatty acids, fatty acyls, prenol lipids, polyketides, and lipids. Lipids have diverse functions in regulating the plasma membrane’s cellular processes and signaling mediation. Plasma membrane lipids are also involved in a plant’s complex interactions with its surrounding microorganisms. Both abiotic and biotic stresses, as well as developmental cues, have long been known to drastically modify lipid composition—including the fatty acid content at the organ level [50]. Here, we discuss the role of polyketides, prenol lipids, and fatty acyls in the innate immunity of *Phlomis purpurea* against *Phytophthora cinnamomi*.

#### 3.2.1. Polyketides

Polyketides represent a family of highly structurally diverse compounds produced by bacteria, fungi, and plants. They have been shown to play important roles, serving as chemical defense agents [51]. β-ketoacyl-acyl carrier protein (ACP) synthases (KS) are involved in the polyketide biosynthesis from units of acyl-CoA [52]. The biosynthesis of polyketides depends on polyketide synthases, which are very diverse in their structure, activity, and mechanism of action. A polyketide synthase has three modules: an acyl-carrier protein (acp), acyltransferase, and malonyl- acp [53]. These modules are well represented in the *P. purpurea* transcriptome. Acyl-acp, β-ketoacyl-acp, enoyl-acp, and transcript profiles are up-regulated at the three time-points (12, 24, and 48 hpc), showing fold change values higher at 12 hpc, decreasing thereafter [25], which agrees with the accumulation profile of polyketides in the *P. purpurea* leaves. Apart from being involved in the biosynthesis of polyketides, β-ketoacyl-acp also plays an important role in cuticle formation. In the case of *P. purpurea*, we cannot exclude the role of these compounds in cuticle formation, since the increased deposition of cuticles is a morphological feature responding to a challenge with the pathogen, as previously demonstrated by Baldé et al., [25]. The role of polyketides in the immune response of *Phlomis purpurea* against *Phytophthora cinnamomi* is also supported by the results of Wang et al. [54], according to whom, the expression of AsPKS1 and *AsPKS2* (polyketide synthases from *Aquilaria sinensis*) are enhanced upon a gibberellins (GA3), MeJA, or SA treatment. This is in agreement with the up-regulation of the transcripts involved in the production of these hormones for some of these time points (GA at 6, 12, 24, and 48 hpc, JA at 6 and 12 hpc, and SA at 6, 12, and 24 hpc), as previously reported by Baldé et al. [25,26]. Our results strongly suggest a protective role of polyketides in *Phlomis purpurea* against *Phytophthora cinnamomi* infection.

#### 3.2.2. Prenol Lipids

Information on the role of prenol lipids in plant biotic stress is scarce. Baczewska-Dąbrowska et al. [55] reported on the role of polyprenols in mitigating salt stress in the leaves of *Tilia x euchlora* trees. According to these authors, this mechanism may be due to the capacity of limiting the transport of Cl^–^ and Na^+^ to leaves. Prenol lipids (coenzyme Q, quinone containing isoprenoid side chains) are essential for energy metabolism in the electron transport system and function as antioxidants within membrane systems [56]. In *Morus alba*, Ackah et al. [57], demonstrated an increase in the prenol lipids in leaves under drought conditions. In *Cycas* plants, under low temperatures, lipids play important roles in membrane structure, signal transduction, and energy storage, which are closely related to the stress responses of plants [58]. Some of the known lipid molecules involved in rhizosphere signaling belong to the categories of fatty acyls, sterol lipids, prenol lipids, saccharolipids, and polyketides [59]. The role of prenol lipids as essential for immune response has been emphasized. In *P. purpurea*, the fold change of prenol is low, mostly negative in the leaves, where it only increases at 48 hpc. In the roots, the maximal fold change is observed at 72 hpc. In combining the data obtained for the *Phlomis purpurea* with those reported by Macabuhay et al. [59], we are tempted to hypothesize that prenol lipids may function as antioxidants and stabilizers of cell membranes after *Phytophthora cinnamomi* aggression. Considering the increased production of these lipids after being challenged and their inverse profiles in the leaves and roots, more studies are needed to better understand the role of prenol lipids in biotic stress.

#### 3.2.3. Fatty Acyls

In *P. purpurea* leaves, fatty acyls present a high fold change (6.7) at 6 hpc, decreasing to negative values from 24 to 72 hpc. The accumulation profile in the roots is opposite. Many studies have pointed out the role of unsaturated fatty acids in plant stress (reviewed by He and Ding [60]). Fatty acids and their degradation products have been considered as inducers/modulators of the plant defense signaling molecules that may regulate a plant’s innate immunity [61]. Taken together, the results on the different types of lipids and their production along the time course of the *Phlomis purpurea* challenge with *Phytophthora cinnamomi*, considering that they represent about 44% and 53% of the total metabolites analyzed in the leaves and roots, respectively, more research should be focused on the different categories to precisely understand their role as a regulatory signal in plant pathogen interactions and plant immunity.

### 3.3. Taurine

Taurine, an organic osmolyte, has received the most attention for its production and role in human diseases. It has been largely assumed that taurine is produced by animals. According to Kataoka and Ohnishi [62], taurine is produced in Rhodophyta, Phaeophyta, Chlorophyta, Fungi, Bryophyta, and Pteridophyta, but in Spermatophyta, it has not been detected at all. The same authors stated that taurine contents are high in lower plants and low in higher plants. Taurine synthesis is highly inducible in freshwater algae and largely constitutive in marine algae, which is consistent with the hypothesis that this compound is involved in osmoregulation [63]. In *Triticum aestivum*, exogenous taurine substantially improved growth, photosynthetic pigments, and nutrient uptake by regulating ROS scavenging, secondary metabolism, and ions homeostasis. The plant was protected from the detrimental effects of boron and chromium through up-regulating of nitric oxide, hydrogen sulfide, glutathione, and phenolic compound production [64]. In tomato in vitro cultures, taurine may counteract the effect of stress caused by the stress factors (NaCl and hydrogen peroxide) and can serve as an effective anti-stress agent in plant cells [65]. According to the author [65], taurine can be used as an anti-stress agent not only for animal systems, but also for plants in general. Fausto et al. [66] reported that, in olive trees growing in managed soils, among other compounds, the up-regulation of taurine may play a pivotal role in protecting plants from biotic and abiotic stress. In a recent paper [67], the potential role of taurine in modulating the defense system of *Trifolium alexandrinum* plants under Mn toxicity has been reported. According to these authors, taurine circumvents Mn-induced oxidative stress by up-regulating the activities of antioxidant enzymes (ascorbate peroxidase, peroxidase, catalase, glutathione-reductase, glutathione-*S*-transferase, and superoxide dismutase) and the levels of ascorbic acid, proline, anthocyanins, phenolics, flavonoids, and glutathione. In the symbiotic algae *Symbiodinium* sp., taurine might affect its metabolic pathways by altering the permeability of the algal cell membrane, diverting photosynthetically fixed carbon from storage compounds to translocated compounds [68]. Taurine protects biological cells, maintains the stability of cell membranes, and protects the activity of antioxidant enzyme systems [69]. It has been reported that taurine accumulation in rice-resistant cultivars (IR56) notably increases during brown planthopper (BPH) infestation and may work toward reducing the ROS-induced oxidative pressure caused by BPH feeding [70]. In *Astragalus membranaceus* var. *mongholicus*, Gao et al. [71] considered taurine to be a resistance-related (RR) metabolite against *Fusarium* root rot. Few reports are available on the function of taurine in plant defense responses against pathogens and the data reported mostly concern its exogenous application. In *P. purpurea,* a high fold change increase is observed at 6 and 12 hpc in its leaves, decreasing thereafter to negative values at 72 hpc. Being the biotic stress responsible for oxidative stress and taking into consideration the *P. purpurea* transcriptome analysis and results reported by Hafeez et al. [67] and Ashraf et al. [64], we are tempted to suggest that taurine may enhance the catalase and peroxidase expression, as well as that of proline, glutathione, and ascorbate, which present high up-regulation levels at 12 hpc, decreasing thereafter, as previously reported [25]. An NMR-based metabolomics study by Gao et al. [71] showed taurine production by *Astraglus membranaceus* var. *mongholicus* against root rot. The transcriptome analysis of *P. purpurea* also revealed very high expression levels of alanine, arginine, aspartate, glutamate, glutamine, phenylalanine, and threonine at 12 hpc. Interestingly, the over-production profile of taurine is similar to that of the antioxidant enzymes identified in the transcriptome analysis of *P. purpurea* [25]. Our results strongly suggest a role of *Phlomis purpurea* constitutive taurine in reducing ROS-induced oxidative bursts very early upon an infection with *Phytophthora cinnamomi,* contributing to the innate immunity of *Phlomis purpurea* against this pathogen. Considering that taurine plays a role in the photosynthesis efficiency in the symbiotic algae *Symbiodinium* sp. [68], we are tempted to also suggest a role of taurine in terpenoids production, by maintaining the chloroplasts’ integrity after a *Phlomis purpurea* challenge with *Phytophthora cinnamomi*.

The constitutive production of putatively identified taurine in *Phlomis purpurea* plants and its up-regulation after challenge with the pathogen *Phytophthora. cinnamomi* account for its inclusion as an RR metabolite naturally occurring in this species. To the best of our knowledge, this is the second report on the natural production of taurine by plants under biotic stress.

Considering the information available and our results, we strongly suggest a main role of taurine in the innate immunity of *P. purpurea.*

### 3.4. Imidazole-4-acetaldehyde

Imidazole-4-acetaldehyde exists in all living organisms, ranging from bacteria to humans. It is a naturally occurring aldehyde derived from histamine by the action of histaminase (E.C. 1.4.3.6) and can be synthesized by the oxidation of histidine [72]. In animals, the oxidative deamination of histidine gives rise to imidazole acetaldehyde [73]. Imidazole represents a building block of histidine and histamine, as well as certain plant alkaloids [74]. The metabolomics workbench includes imidazole-4-acetaldehyde in the alkaloids superclass and histidine alkaloids main class.

Recently, Yang et al. [75] reported on the presence of imidazole-4-acetaldehyde in *Capparis spinosa* fruit and its role in protecting against acute liver injury. Imidazole is present in the structures of several key molecules of major biological significance, most notably purines and histidine [76].

The data from the *Phlomis purpurea* transcriptome, revealing the high expression levels of the transcripts involved in histidine and purine biosynthesis, combined with the metabolome data for imidazole-4-acetaldehyde, suggest an important role of imidazole-4-acetaldehyde in the immune response of *Phlomis purpurea* to *Phytophthora cinnamomi*. Tolomeu and Fraga [77] reported on the importance of imidazole-based compounds in the maintenance of life. They also emphasized the role of imidazole compounds as antifungals and antioxidants in animals. Considering that the oxidative deamination of histidine gives rise to imidazole acetaldehyde [73], and that imidazole represents a building block of alkaloids [74], the profiles of imidazole alkaloids in *Phlomis purpurea* leaves after a challenge suggest an important role of this imidazole alkaloid in the innate immunity of *Phlomis purpurea* against *Phytophthora cinnamomi.* To the best of our knowledge, this is the first time that the overproduction of imidazole-acetaldehyde has been reported following an infection with a pathogen. Further studies will clarify the role of this metabolite in innate immunity.

### 3.5. Alkaloids

Alkaloids can act as defense compounds in plants, being efficient against pathogens and predators due to their toxicity. The fast perception of aggressors and unfavorable environmental conditions followed by an efficient and specific signal transduction for triggering alkaloid accumulation are key steps for successful plant protection [78]. The metabolome analysis of *Phlomis purpurea* after being challenged with *Phytophthora cinnamomi* revealed the production of ornithine, tyrosine, and tryptophan alkaloids, with different production levels at different times post-challenge. The opposite profiles obtained for the leaves and roots suggest their translocation from leaves to roots, where they may contribute to protecting against the root pathogen *Phytophthora cinnamomi*. Specialized metabolites such as alkaloids are found in varying levels in the different tissues of plants (e.g., leaf and root) and offer chemical protection against a diverse variety of pests or pathogens [79]. Crossing the data from the transcriptome and metabolome analyses, a close correlation can be established between the up-regulation of tyrosine, tryptophan, and ornithine transcripts and the biosynthesis of the alkaloids putatively identified in the metabolome analysis of *Phlomis purpurea*. As for the other metabolites discussed, a role of these alkaloids in the protection against *P. cinnamomi* can be suggested. Our results agree with those of Munir et al. [80] on *Gerbera hybrida*, pointing out the role of the tyrosine metabolic pathway in response to the *Phytophthora cryptogea* root-rot-causing agent.

Alkaloids play a very important role in the immune systems of animals and plants [81]. In *Phlomis purpurea*, alkaloids may be part of the over-produced secondary metabolites that justify the immunity of this species against *Phytophthora cinnamomi*, as suggested by the up-regulation of transcripts reported by Baldé et al. [25].

### 3.6. Flavonoids and Phenylpropanoids

Flavonoids are a family of phenolic compounds with strong antioxidant activity present in fruits, vegetables, and other plant foods, produced by vascular plants [82]. Flavonoids have been assigned a role as phytoalexins. Their antibacterial activity has been intensively studied due to the interest in finding new phytochemical compounds with pharmacological activities (reviewed by Farhadi et al. [83] and Song et al. [84]). Nine antifungal flavonoids were isolated from adzuki bean (*Vigna angular*) roots treated with the pathogenic fungus, *Cephalosporium gregatum* [85]. In *P. purpurea*, the flavonoids content is high in the roots throughout the challenge time and decreases in the leaves from 48 hpc, while increasing in the roots. Within the cell, chalcone isomerase is involved in the synthesis of flavonoids [86]. In *Phlomis purpurea* challenged with *Phytophthora cinnamomi*, the high up-regulation of chalcone isomerase after a challenge [25] accounts for the increased production of this secondary metabolite.

Flavonoids may have the potential to scavenge the ROS generated after the contact of the pathogen with *P. purpurea.* According to Agati et al. [87], ‘antioxidant’ flavonoids are found in the chloroplast, which suggests their role as scavengers of singlet oxygen and stabilizers of the chloroplast outer membrane envelope, with this role being corroborated by Mierziak et al. [88].

Flavonoid localization and synthesis in different cell types and in response to environmental stimuli can be regulated by several transcription factors, in particular those of the MYB and bHLH families [89,90]. The MYB TF family is one of the largest families of transcription factors that regulate the flavonoid biosynthesis in plants [30]. It has been reported that the R2R3-MYB gene *SbMYB8* from *Scutellaria baicalensis* regulates flavonoid biosynthesis through the binding of the SbMYB8 protein to the GmMYB92 BS3 sequence in the SbCHS promoter region [91]. As reported before, a high up-regulation of both *R2R3-MYB* and *MYB8*, as well as of *bHLH* transcription factors, was observed after the challenge of *Phlomis purpurea* with *Phytophthora cinnamomi* [25,26]. Flavonoids are likely to move via vesicle-mediated transport or through MATE (multidrug and toxic extrusion compound) families [92]. MATE transporters have a substrate preference for flavonoids with diverse chemical structures [93]. It is interesting to notice the up-regulation of *MATE* transcripts in *P. purpurea* for the same time points, which may account for the role of these transcripts in flavonoid transport to the vacuole.

Plant phenylpropanoids are a vast and structurally diverse group of phenylalanine-derived metabolites (C_6_–C_3_) that play crucial roles in a plant’s interaction with other living organisms [94]. The profiles of phenylpropanoids in *P. purpurea* leaves are different from those in roots. While in the leaves they decrease to negative values from 6 hpc, in the roots they increase to high fold change between 24 and 48 hpc, decreasing to negative values at 72 hpc. Using a metabolome analysis to understand the chemical defense of cereals against the pathogen *Fusarium graminearum*, Gauthier et al. [95] have demonstrated the interplay of phenylpropanoids, terpenoids, and fatty acid derivatives in the defense against this pathogen.

In a recent review, Dong and Lin [96] pointed out that phenylpropanoid metabolism is modulated by diverse signaling pathways and multiple regulation mechanisms, including transcriptional and post-transcriptional regulation, phytohormone signaling pathways, and biotic and abiotic stresses. According to these authors, the transcriptional regulation of phenylpropanoid metabolism by MYB, R2R3-MYB, and bHLH TFs is believed to be crucial for lignin and flavonoid biosynthesis and the overall control of phenylpropanoid biosynthesis. Phenylpropanoid compounds can perform or induce physical and chemical barriers against pathogen infection, as well as the secretion of signal molecules that can ultimately induce defense-related gene expression in plants [97]. Our results from the transcriptome analysis of *Phlomis purpurea* after being challenged with *Phytophthora cinnamomi* demonstrated a high up-regulation of the mentioned transcription factors, as well as of cinnamic acid 4-hydroxylase and 4-coumarate-3 hydroxylase [25,26]. These results allied to the over-production by *Phlomis purpurea* of constitutive phenylpropanoids after challenging with the pathogen account for a role of these metabolites in *Phlomis purpurea* resistance to *Phytophthora cinnamomi*, functioning as plant defense mediators and in cell wall lignification.

## 4. Concluding Remarks

Pest and pathogen attacks on woody fruit or forest tree species are increasing due to global changes and constitute a serious problem worldwide, causing severe diseases and economic losses. The resistance of *Phlomis purpurea* to the oomycete *Phytophthora cinnamomi* may result from the interplay of multi-compound metabolite classes and the corresponding expressions of genes coding for the enzymes participating in different metabolic pathways. An interesting feature of our study is the constitutive production of taurine and imidazole-4-acetaldehyde and their increased content after a challenge with *Phytophthora cinnamomi*. To the best of our knowledge, we report for the first time the roles of taurine and imidazole-4-acetaldehyde in the plant innate immunity of *Phlomis purpurea* against the oomycete *Phytophthora cinnamomi*.

Our results on taurine and imidazole-acetaldehyde suggest that they may likely serve as candidate biomarkers capable of discriminating plant resistance levels and may be used to identify the species and varieties better suited to cope with fungi or other plant pathogen diseases. The knowledge on the taurine metabolic pathway and its corresponding genes may constitute an important contribution for improving plants’ resistance towards pathogens.

The understanding of the *Phlomis purpurea* response to the biotic stress imposed by the oomycete *Phytophthora cinnamomi* brings additional knowledge about the mechanisms related to plant defense against pathogens and opens up perspectives for the use of this plant as a strategy safer than the use of fungicides or other hazardous molecules, in order to prevent the root rot of crops or forest species growing together with *P. purpurea*.

## 5. Materials and Methods

### 5.1. Plant Material

The production, growth, and maintenance of the experimental plants were achieved as previously described [23]. Briefly, *Phlomis purpurea* seeds were collected in the field across the Algarve in southern Portugal. They were stored at 4 °C until they were processed. Prior to germination, the seeds were surface sterilized and rinsed in sterile distilled water. The moistened seeds were placed between two absorbent paper discs in Petri dishes until germination occurred. When the radicles were 2–3 cm long, they were transferred into cylindrical soft black plastic tubes (25 cm × 3 cm) containing vermiculite.

### 5.2. Phytophthora cinnamomi Isolates

Pure stock cultures of the *P. cinnamomi* isolates, PA37 and PA45, both mating type A2, were isolated in the Algarve region (southern Portugal) from *Quercus suber* roots from Lagos and soil associated with declining *Q. suber* stands in S. Brás de Alportel, respectively. The isolation and culture maintenance took place on V8 Juice agar medium, as described by Horta et al. [98].

### 5.3. Zoospore Production

Zoospores were produced following a modification of the procedure reported by Byrt and Grant [99] and described by Baldé et al. [25]. Briefly, a *P. cinnamomi* culture plug was transferred onto 10% V8 juice agar medium (V8A) and incubated for 3 days at 24 °C. The V8A plugs from the growing colony in the Petri dishes were transferred to Miracloth membranes. The cultures were incubated for 15 days at 24 °C. The Miracloth support and mycelia were transferred to 100 mL of 5% V8 broth (V8B) and the culture was shaken overnight (16 h) at 90 rpm at 24 °C. The nutrient medium was replaced with a solution (MSS) consisting of 0.01 M Ca(NO_3_)_2_·4H_2_O, 0.005 M KNO_3_, and 0.004 M MgSO_4_·7H_2_O, dissolved in 1 L of distilled water, autoclaved, and subsequently supplemented with 1 mL of 0.1 M ferric sodium EDTA (C_10_H_12_N_2_NaFeO_8_) solution. The culture was then shaken for 24 h. Sporangia were induced to release zoospores by incubating the Miracloth covered with the MSS in Petri dishes at 4 °C for 20 min. Then, the Petri dishes were exposed to fluorescent light at room temperature for 3 h. The zoospore suspension from each Miracloth was transferred into a 15 mL conical tube. The upper 2 mL was transferred to a second tube and shaken for 70 s to encyst the zoospores. In total, 10^4^–10^5^ zoospores mL^−1^ were routinely produced.

### 5.4. Challenging with Zoospores

Two-and-a-half-month-old seedlings obtained from the *P. purpurea* seeds were carefully removed from the vermiculite via immersion in water to wash out the vermiculite from the root system. The intact seedlings were placed in distilled water at 22 °C and challenged immediately by a root dip into 50 mL of a 10^4^ mL^−1^ zoospore suspension in MSS for 0, 6, 12, 24, 48, and 72 h. The controls were made with MSS at the same time point. The plants and controls were kept in glass tubes in the dark at 22 °C. At each time point, the whole plants were harvested, immediately frozen in liquid nitrogen, and kept at −80 °C.

Five plantlets for each time point were pooled. Five replications of the whole experiment were carried out (five replicates).

### 5.5. Metabolite Extraction

*Phlomis purpurea* roots and leaves (10 mg fresh weight) were macerated with 200 μL of MeOH in a 1 mL Wheaton tissue grinder. The samples were transferred into a 1.5 mL Eppendorf tube and 100 μL of MeOH from the grinder washing was added. They were centrifuged at 10,000× *g* for 10 min. The supernatant was recovered, the samples were dried overnight in a Speed Vac (Savant, SC 110A, Holbrook, AZ, USA), and resuspended in 200 μL of MeOH 50% in water.

### 5.6. Untargeted Metabolomic Analysis by UPLC-MS

The samples were again centrifuged at 10,000× *g* for 10 min and analyzed using LCMS on a Waters nanoAcquity UPLC coupled with a nanoESI source (Waters NanoLock Zspray) in positive polarization mode and a Waters Synapt G2 HDMS mass spectrometer. The reversed phase chromatography was performed on a capillary spray tip column (0.2 mm × 50 mm) packed with HSS T3 1.7 mm (Waters). A binary gradient (eluent A, 5 mM ammonium acetate in water; eluent B, 5 mM ammonium acetate in isopropanol acetonitrile (9:1)) was applied, starting from 100% eluent A to 2% A at 10 min, and a flow ramp starting from 2.5 μL min^−1^ to 2.0 μL min^−1^ at 10 min, followed by a hold time of 13 min, resetting to 100% A within 1 min and an equilibration to the initial conditions during 5 min at 2.0 μL min^−1^, totaling 29 min of running time. The injection volume was 1 μL. The nanoESI source was operated at +3.4 kV and a temperature of 100 °C, sample cone voltage of 40 V, and extraction cone voltage of 4 V. The spectra were acquired over 50–2000 *m*/*z* in MS^E^ resolution mode at a rate of 3 Hz in continuum format. Leucine enkephalin was used as an internal calibrant.

### 5.7. Data Analysis and Compound Annotation

The LC-MS CDF files (raw data) were processed with the ‘cosmiq’ R package (https://doi.org/10.18129/B9.bioc.cosmiq, accessed on 30 May 2022) with the settings of RTinfo = TRUE, rtcombine = c(290,750), mzbin = 0.003, continuum = TRUE, profStep = 1, and SNR.Th = 50. Using the ‘MetaboAnalystR’ package (https://doi.org/10.1093/bioinformatics/bty528, accessed on 30 May 2022), the data were batch corrected using the ComBat algorithm to remove any bias. Missing values were substituted by half of the minimum intensity value found within the data. The data were filtered using the “mean” option and normalized by the median. The intensity data were log transformed and auto scaled prior to the multivariate analysis. For each time point, the default parameters of the ‘MetaboAnalystR’ package were used to calculate the fold change between the control and inoculated samples and to perform the multivariate analysis, a partial least squares discriminant analysis (PLS-DA).

Using the ‘MetaboShiny’ metabolomics analysis package [100], the ions, i.e., the *m*/*z* values detected, were putatively annotated and classified against the KEGG database [101]. When a compound was putatively annotated as a lipid, the LIPID MAPS classification was considered [49,102,103].

All the putatively identified ions, i.e., the *m*/*z* values with KEGG identifiers containing KNApSAcK [104] equivalencies, were searched for their presence in the Plantae Kingdom. For compounds with multiple annotations, a manual curation was performed, and compounds only belonging to the Plantae Kingdom were considered. The putative compounds identification and annotation for the leaves and roots at all the time points are represented in Supplementary Tables S1 and S2, respectively.

## Figures and Tables

**Table 1:** Number of differentially accumulated *m*/*z* values and differentially produced putatively identified compounds by LC-MS/MS in leaves and roots of *Phlomis purpurea* at 6, 12, 24, 48, and 72 h post challenge (hpc) with *Phytophthora cinnamomi*.

	Differentially Abundant Ions		Putatively Identified Metabolites	
Time Point	Leaf	Roots	Leaf	Roots
6 hpc	25	48	11	7
12 hpc	17	40	8	11
24 hpc	39	10	13	0
48 hpc	35	30	11	10
72 hpc	18	19	13	6
Total	134	147	56	34

## Data Availability

The data presented in this study are available in the Supplementary Material of the present article.

## Supplementary Materials

The following supporting information can be downloaded at: https://www.mdpi.com/article/10.3390/plants12101929/s1, Figure S1: Combined mass spectra of metabolites from leaves and roots samples of *Phlomis purpurea* non-challenged and challenged with *Phytophthora cinnamomi*; Table S1: Metabolites identified in *Phlomis purpurea* leaves upon challenge with *Phytophthora cinnamomi*; Table S2: Metabolites identified in *Phlomis purpurea* roots upon challenge with *Phytophthora cinnamomi;* Table S3: Differentially accumulated metabolites in *Phlomis purpurea* leaves upon challenge with *Phytophthora cinnamomi*; Table S4: Differentially accumulated metabolites in *Phlomis purpurea* roots upon challenge with *Phytophthora cinnamomi.*
