# Supplementary material for: A Metabolome Analysis and the Immunity of Phlomis purpurea against Phytophthora cinnamomi
Source: Plants (Basel). 2023 May 9;12(10):1929. doi: 10.3390/plants12101929 (PMC10223286; doi:10.3390/plants12101929)

## Slide 1
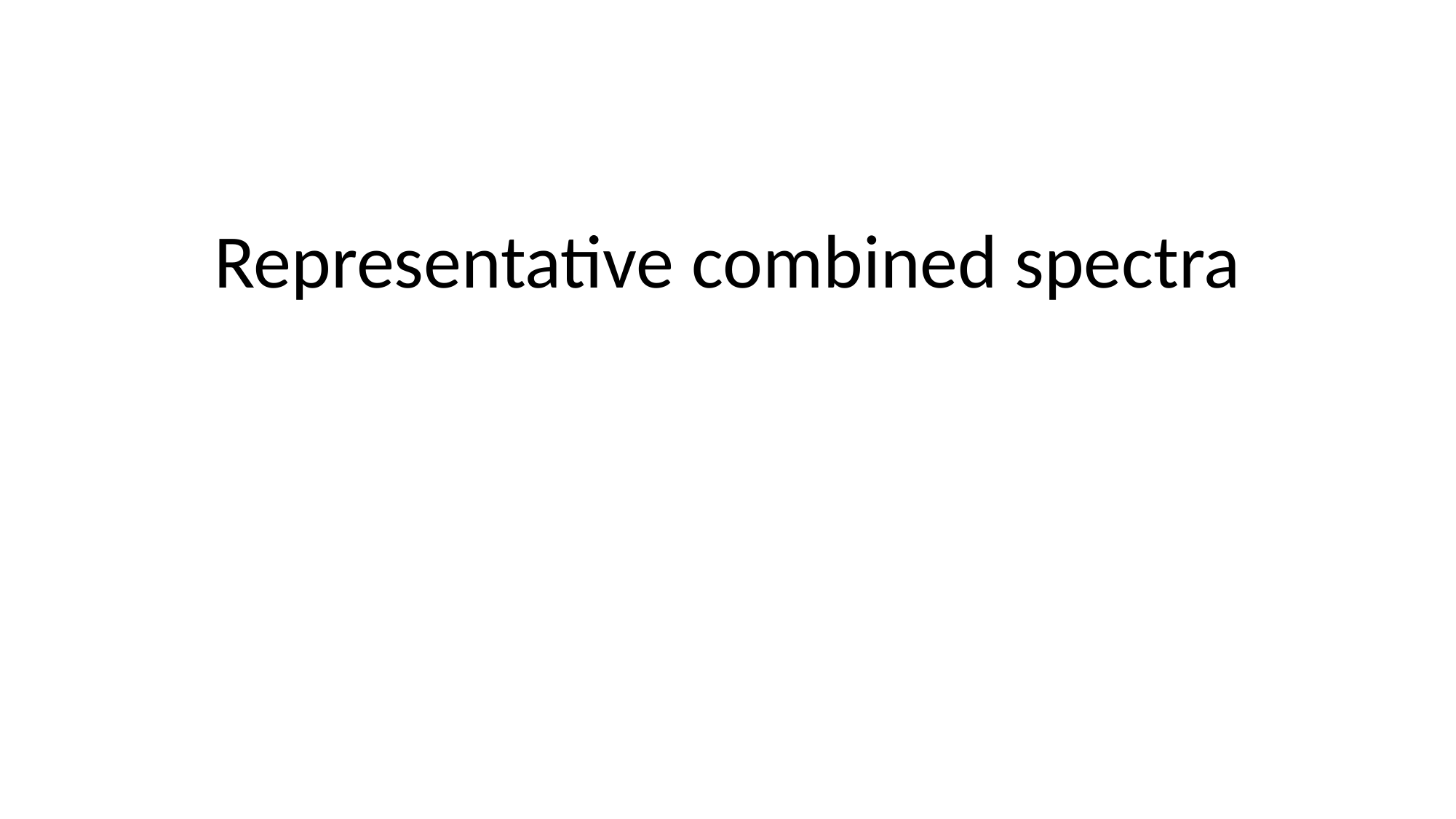

# Representative combined spectra

## Slide 2
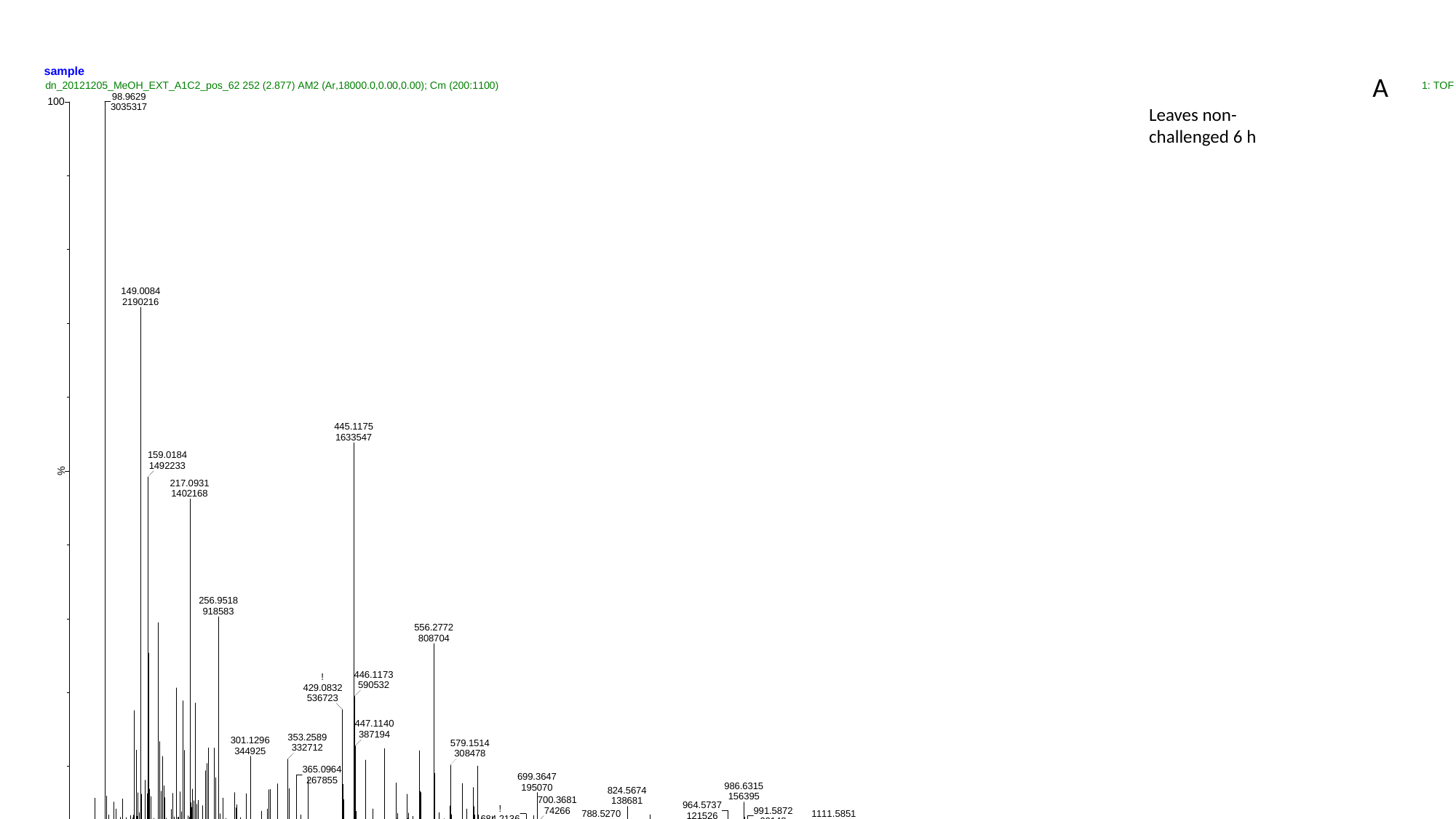

A
Leaves non-challenged 6 h

## Slide 3
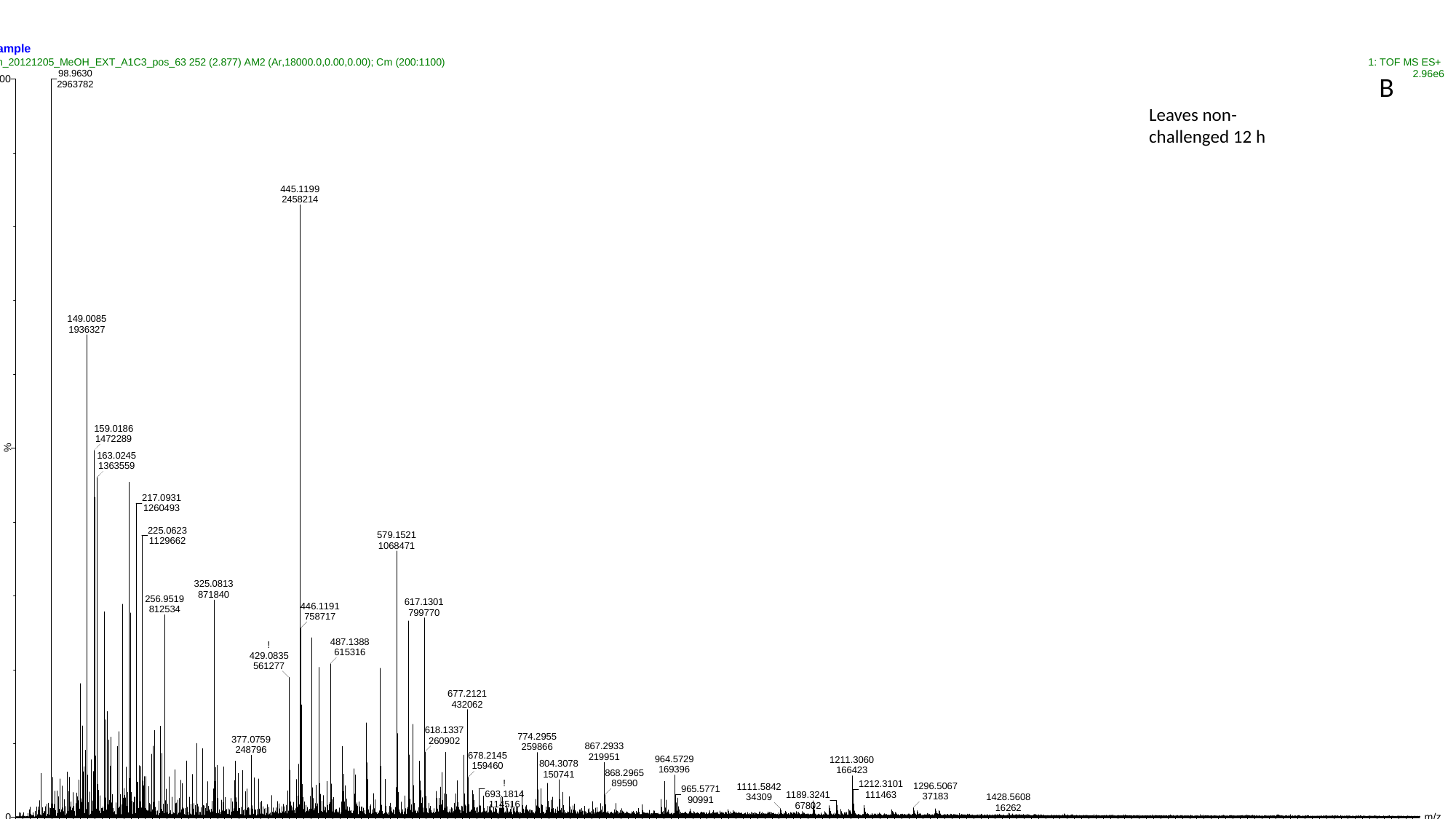

B
Leaves non-challenged 12 h

## Slide 4
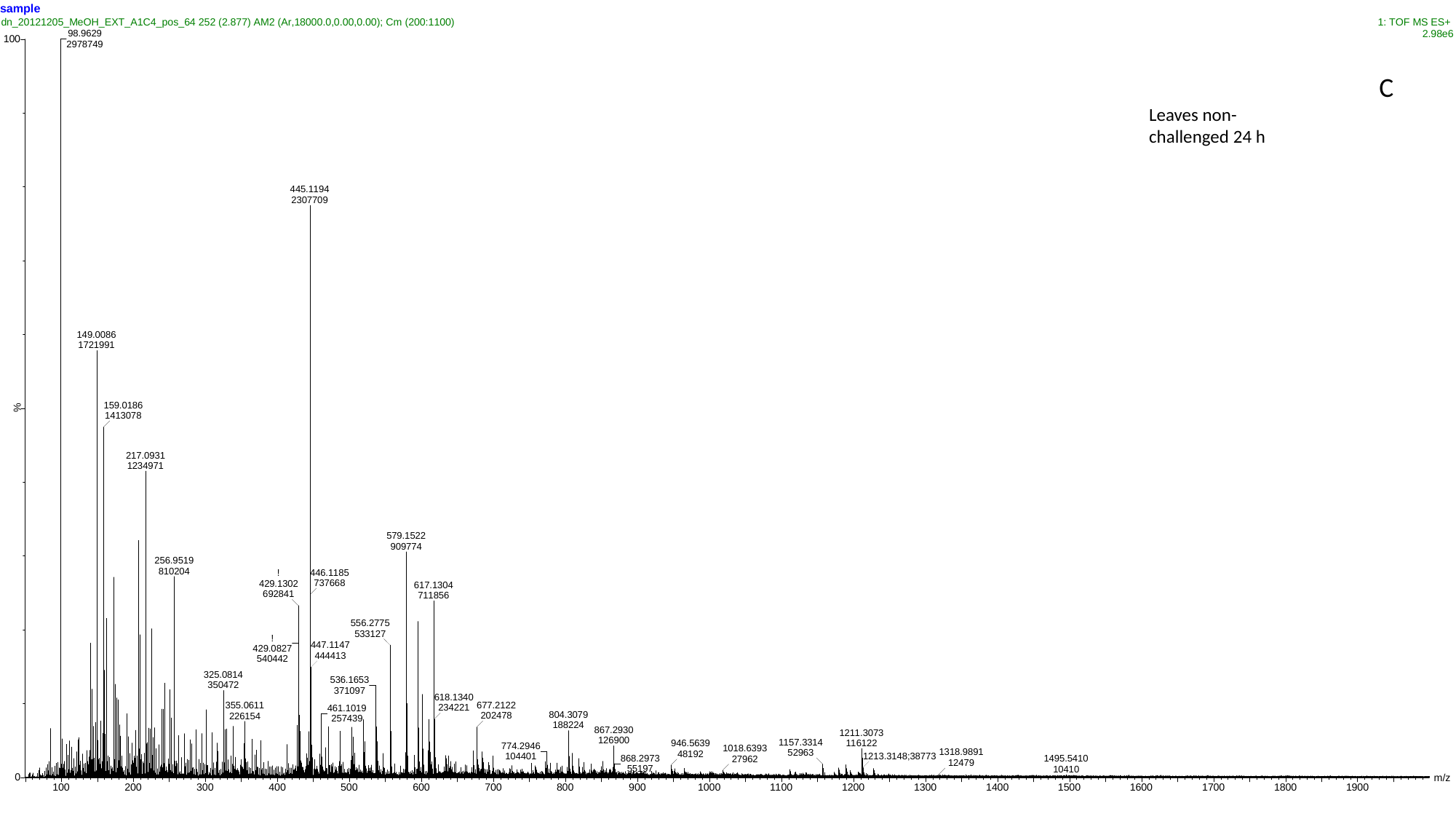

C
Leaves non-challenged 24 h

## Slide 5
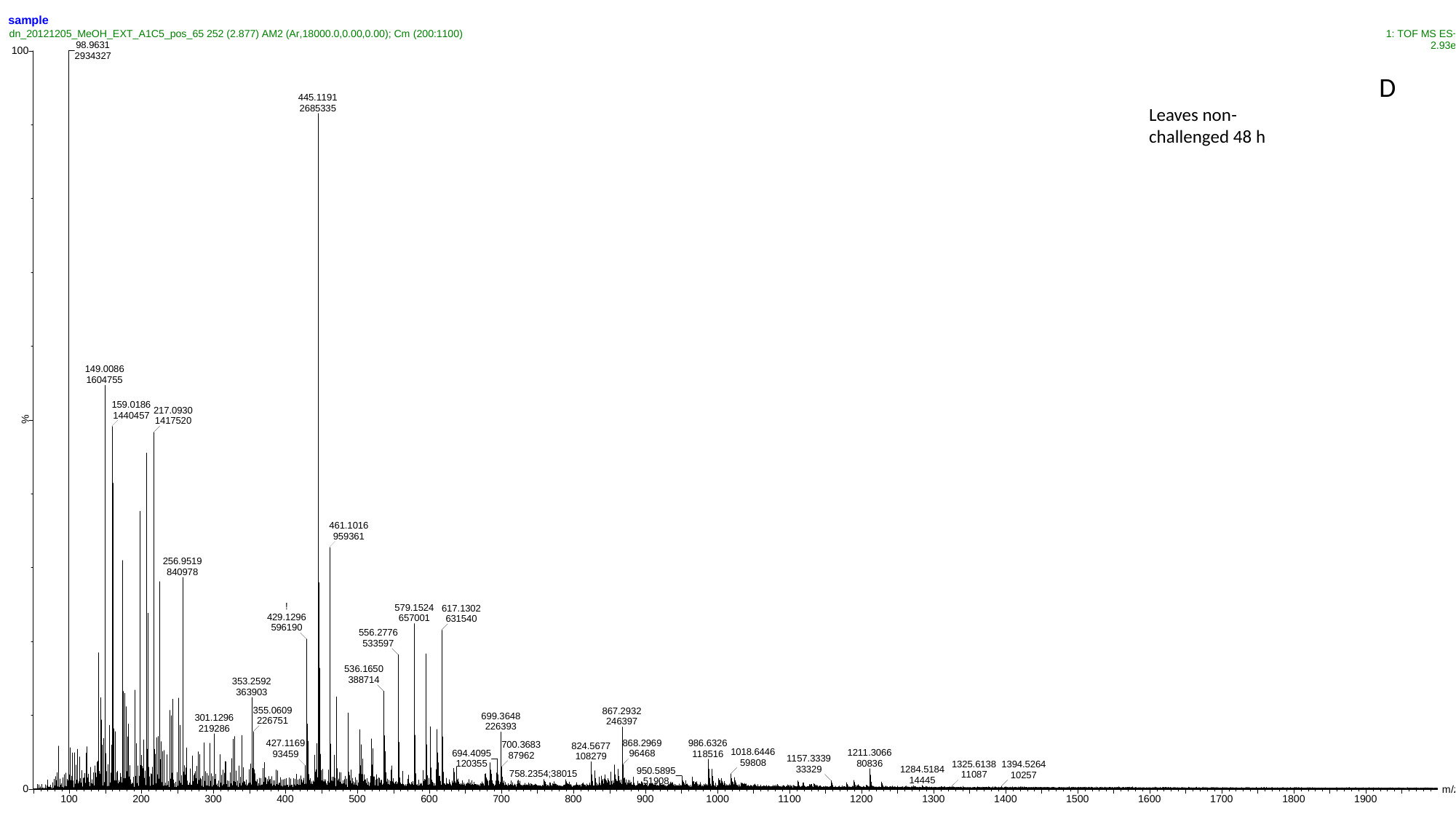

D
Leaves non-challenged 48 h

## Slide 6
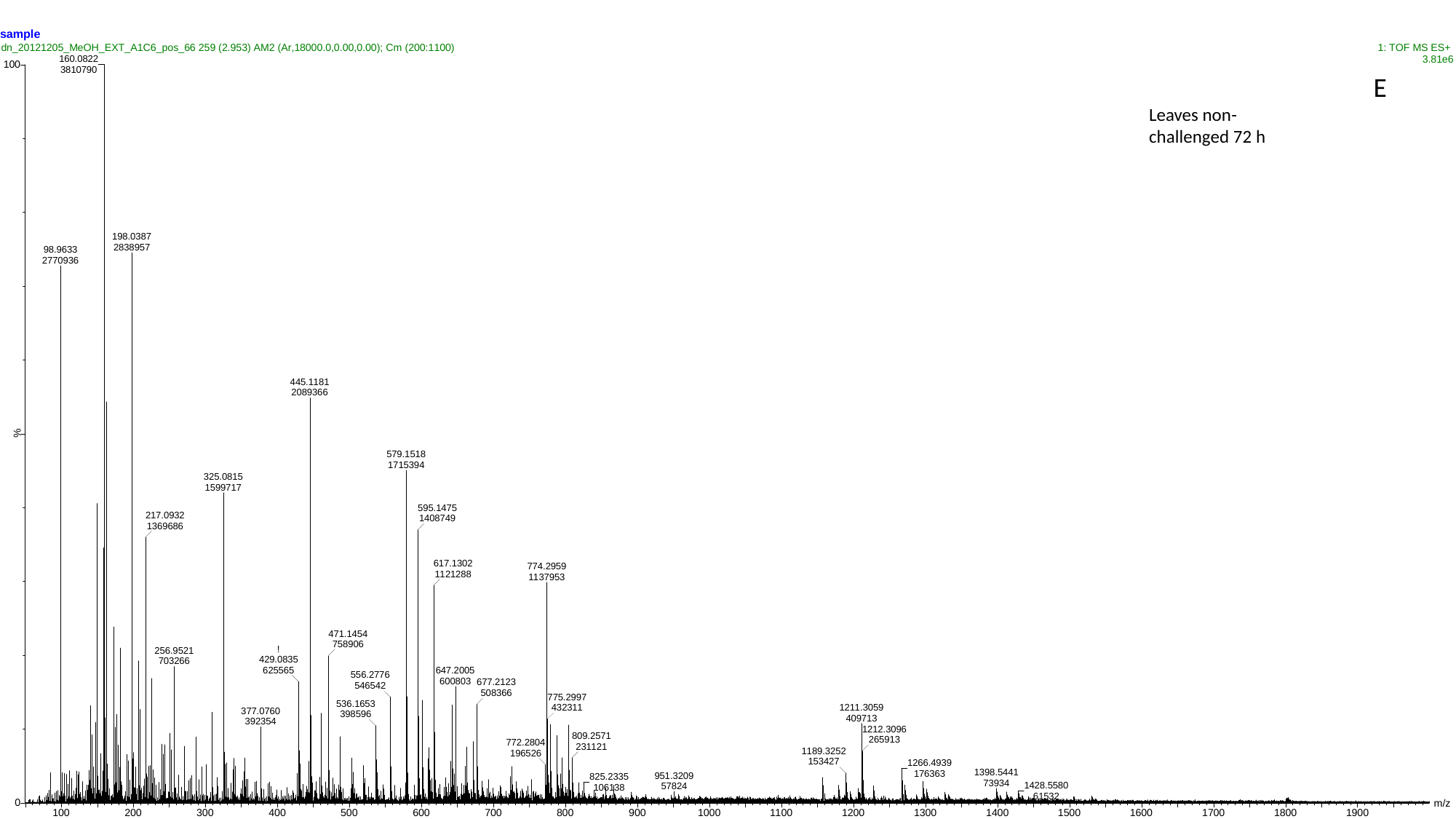

E
Leaves non-challenged 72 h

## Slide 7
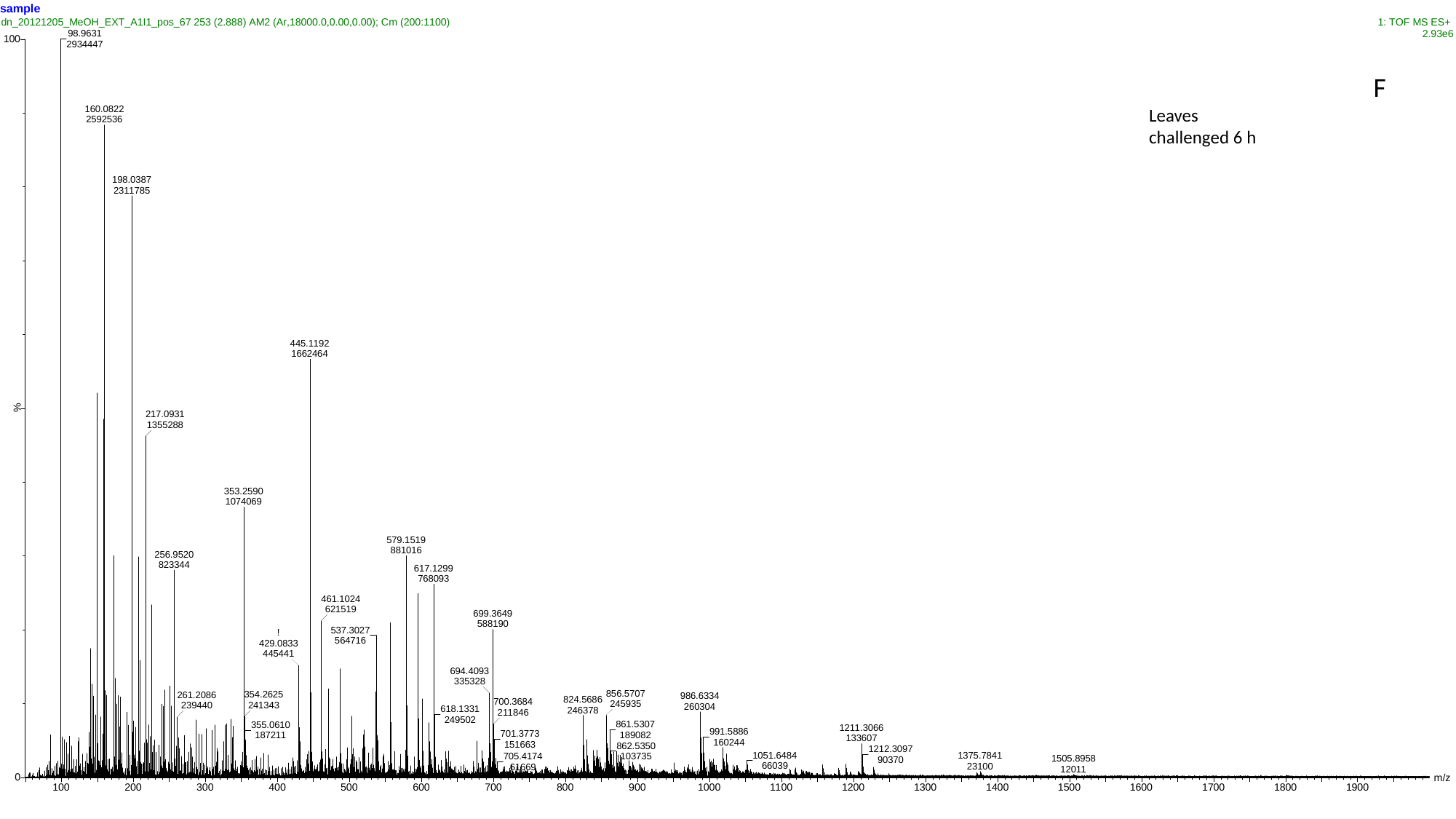

F
Leaves
challenged 6 h

## Slide 8
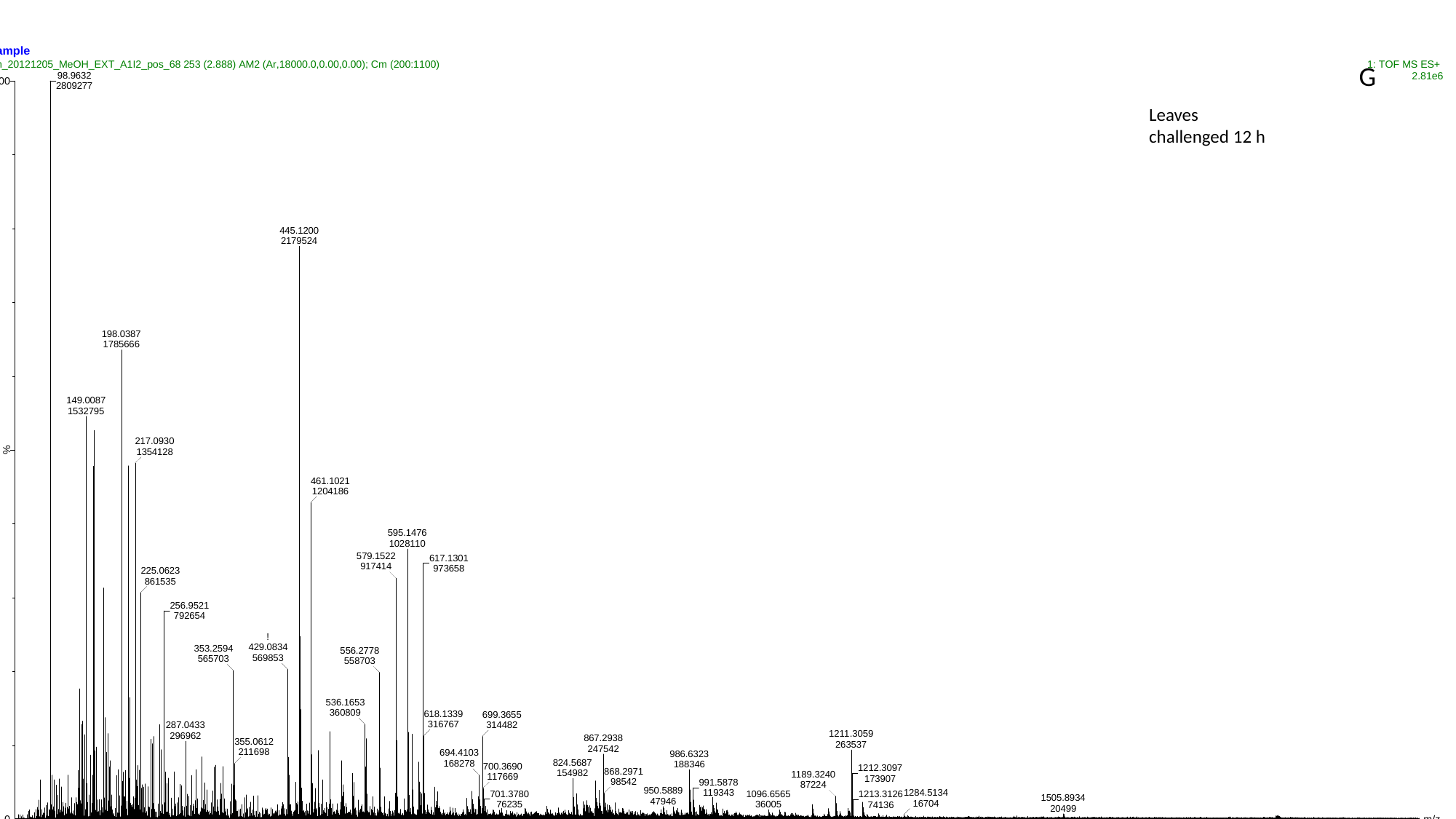

G
Leaves
challenged 12 h

## Slide 9
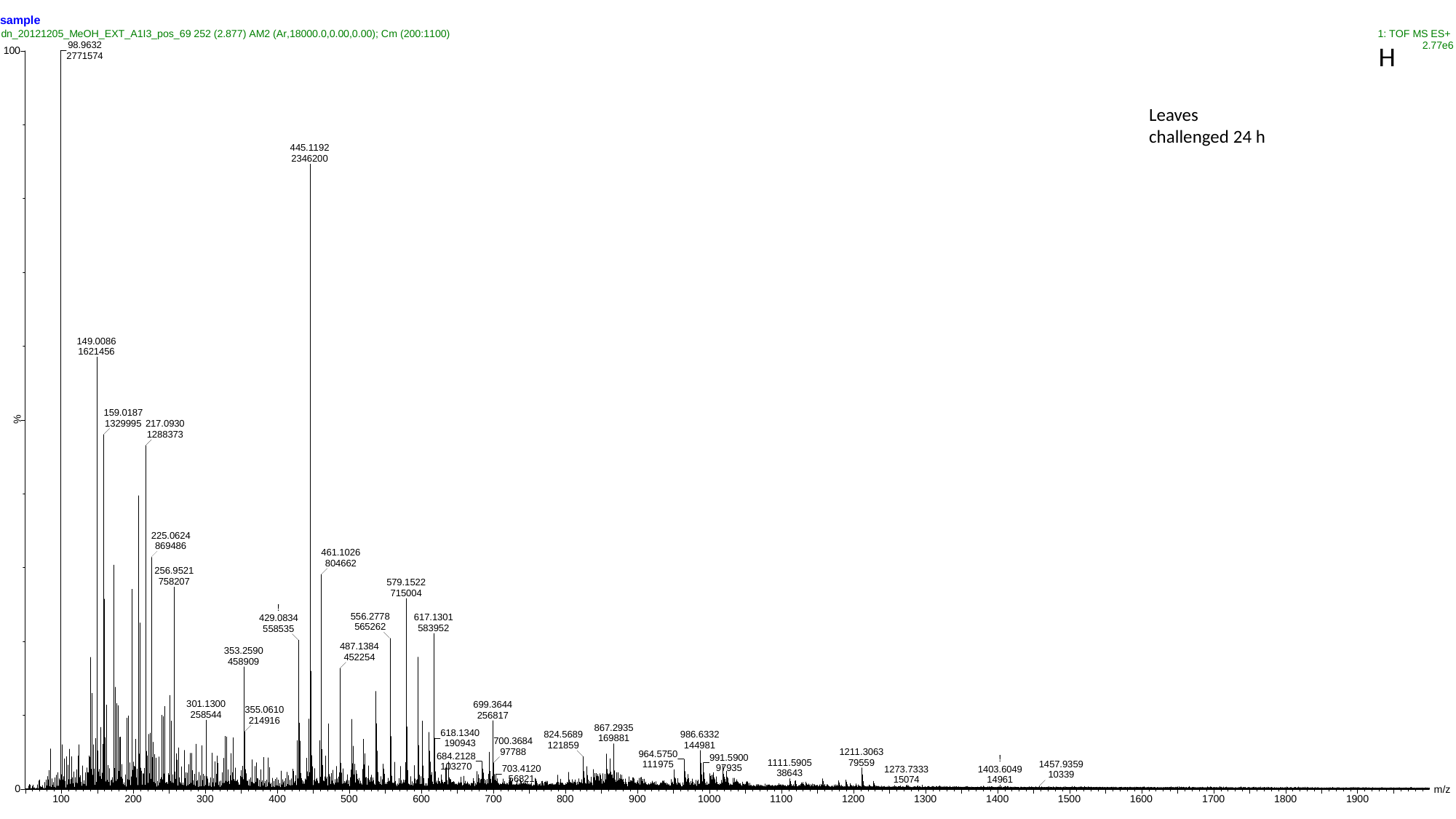

H
Leaves
challenged 24 h

## Slide 10
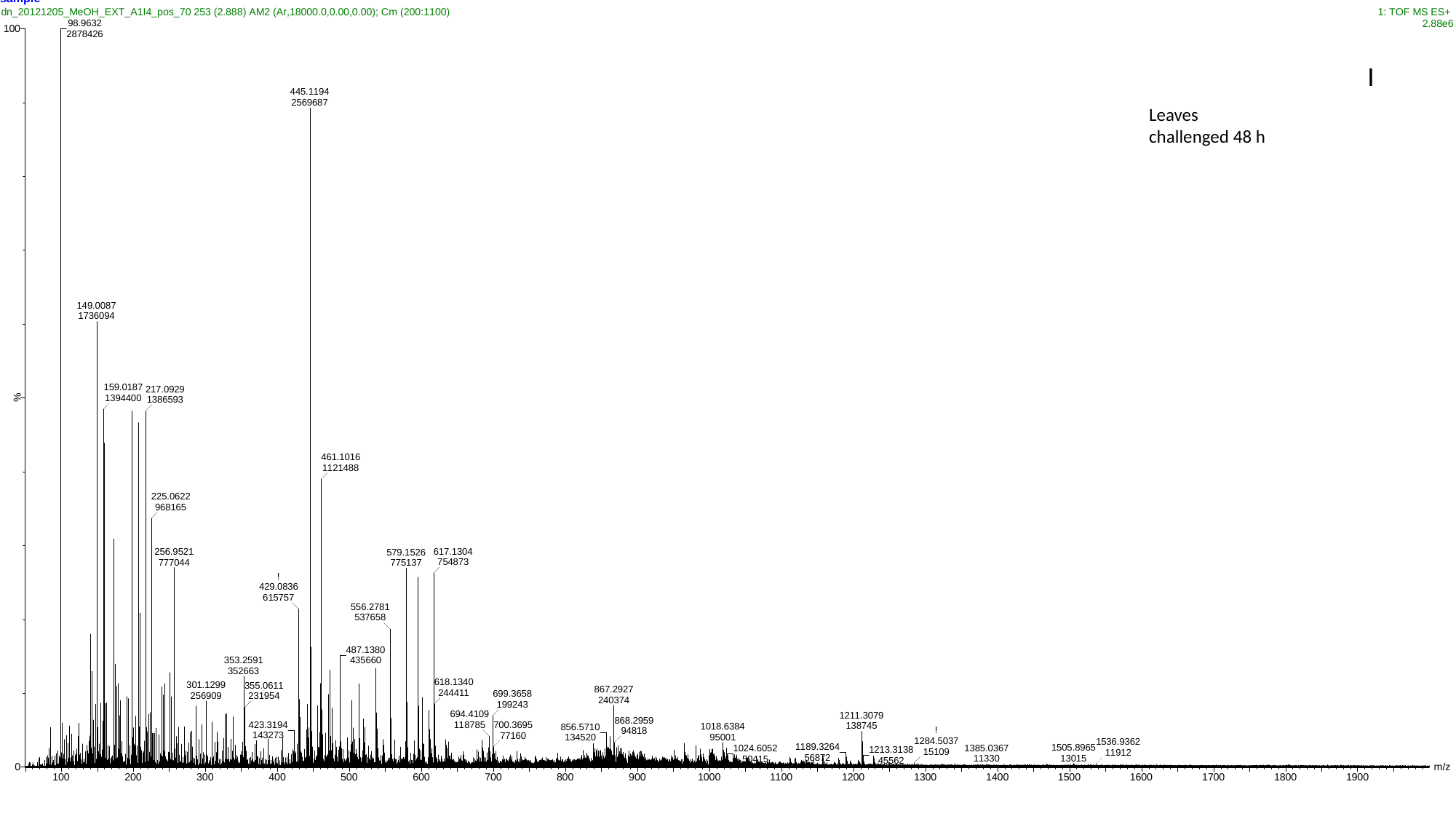

I
Leaves
challenged 48 h

## Slide 11
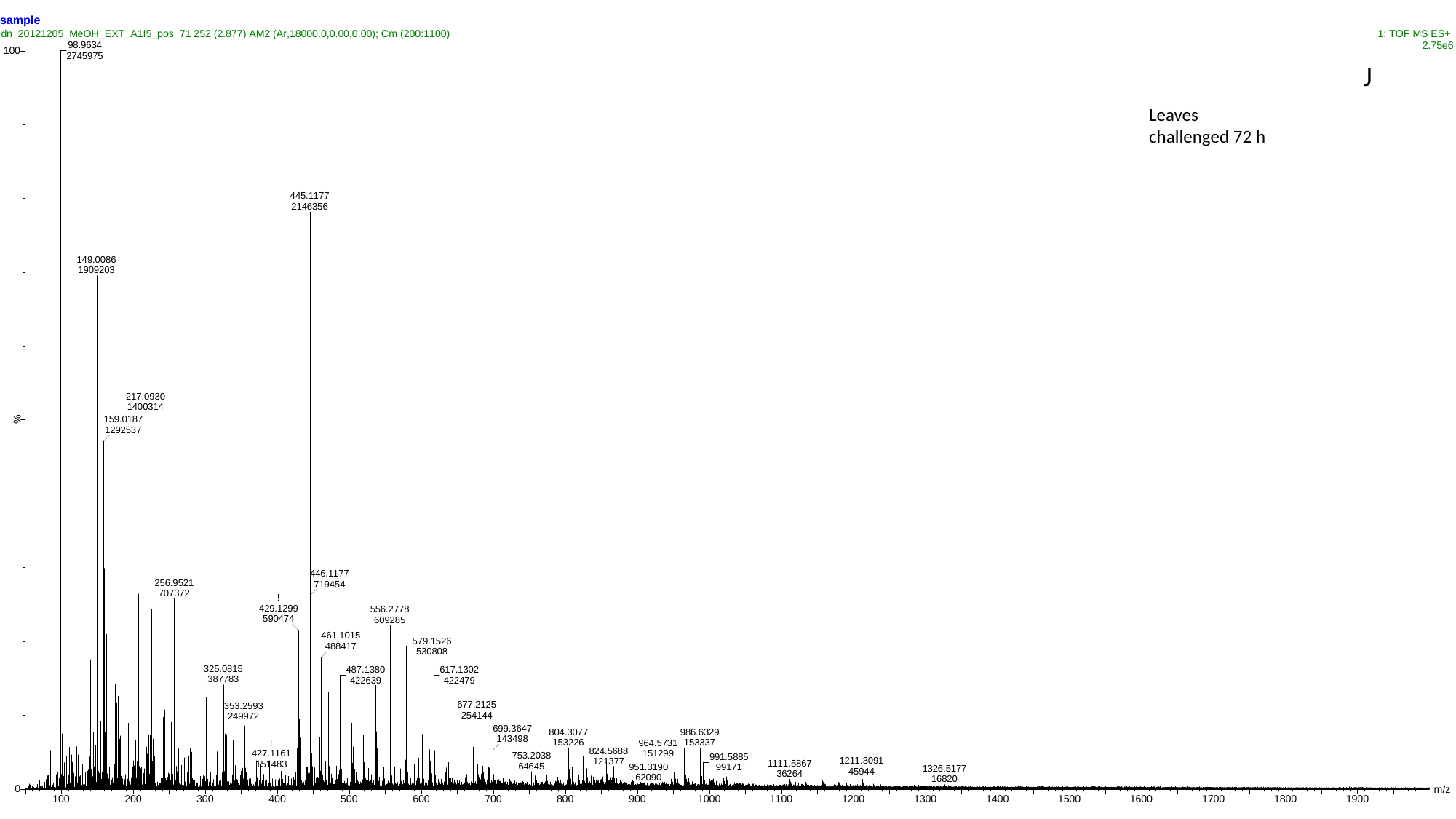

J
Leaves
challenged 72 h

## Slide 12
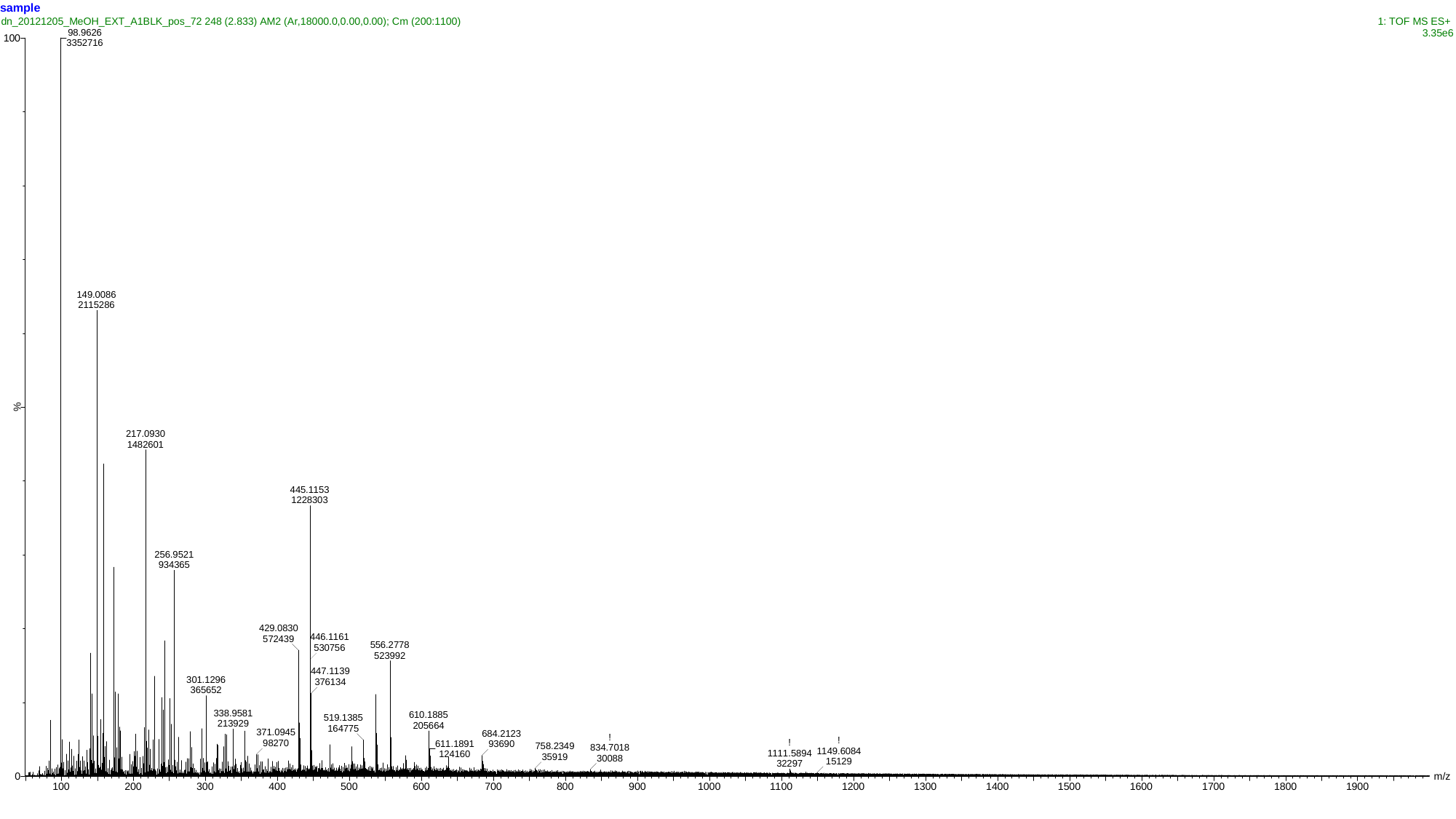

## Slide 13
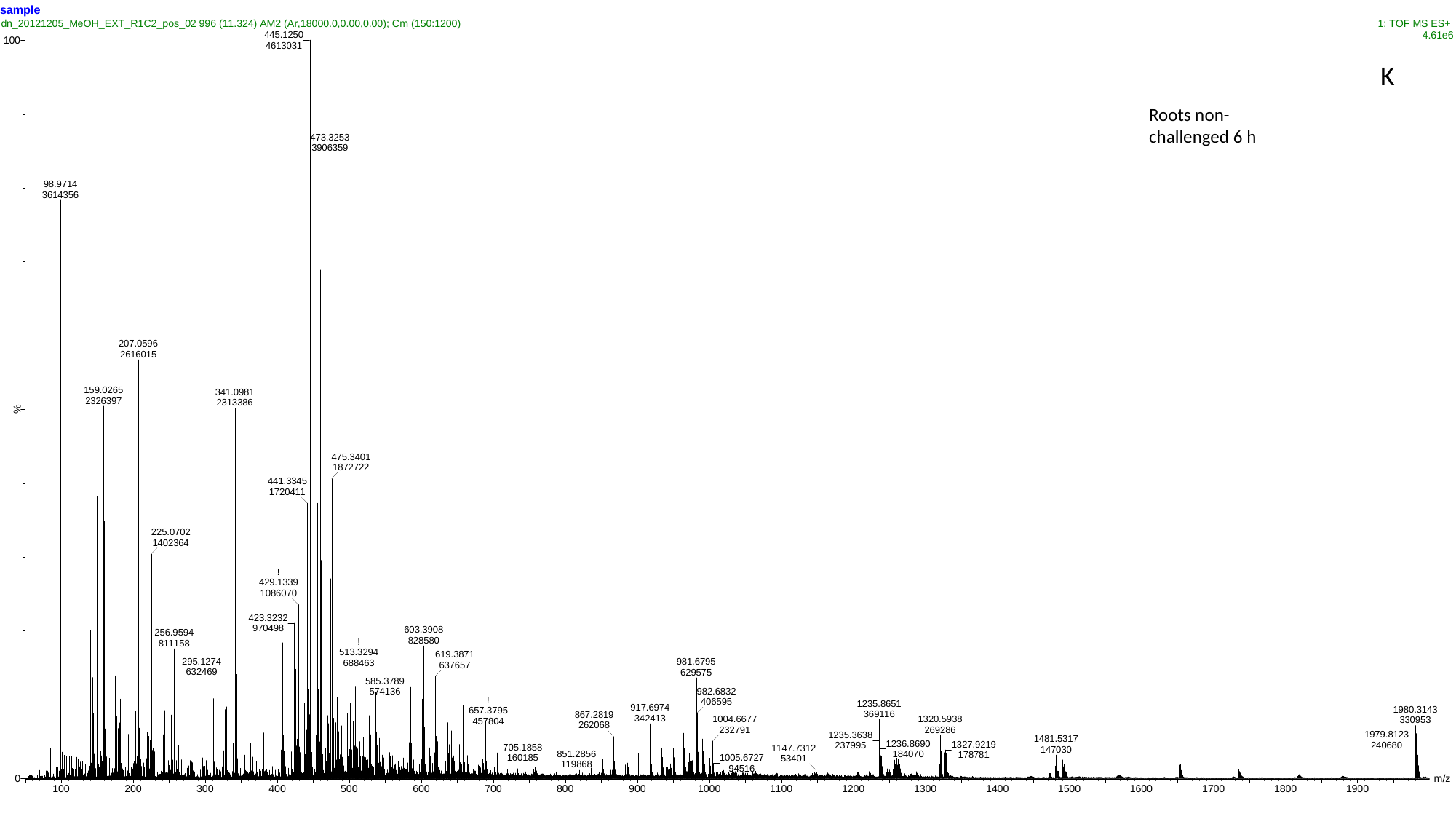

K
Roots non-challenged 6 h

## Slide 14
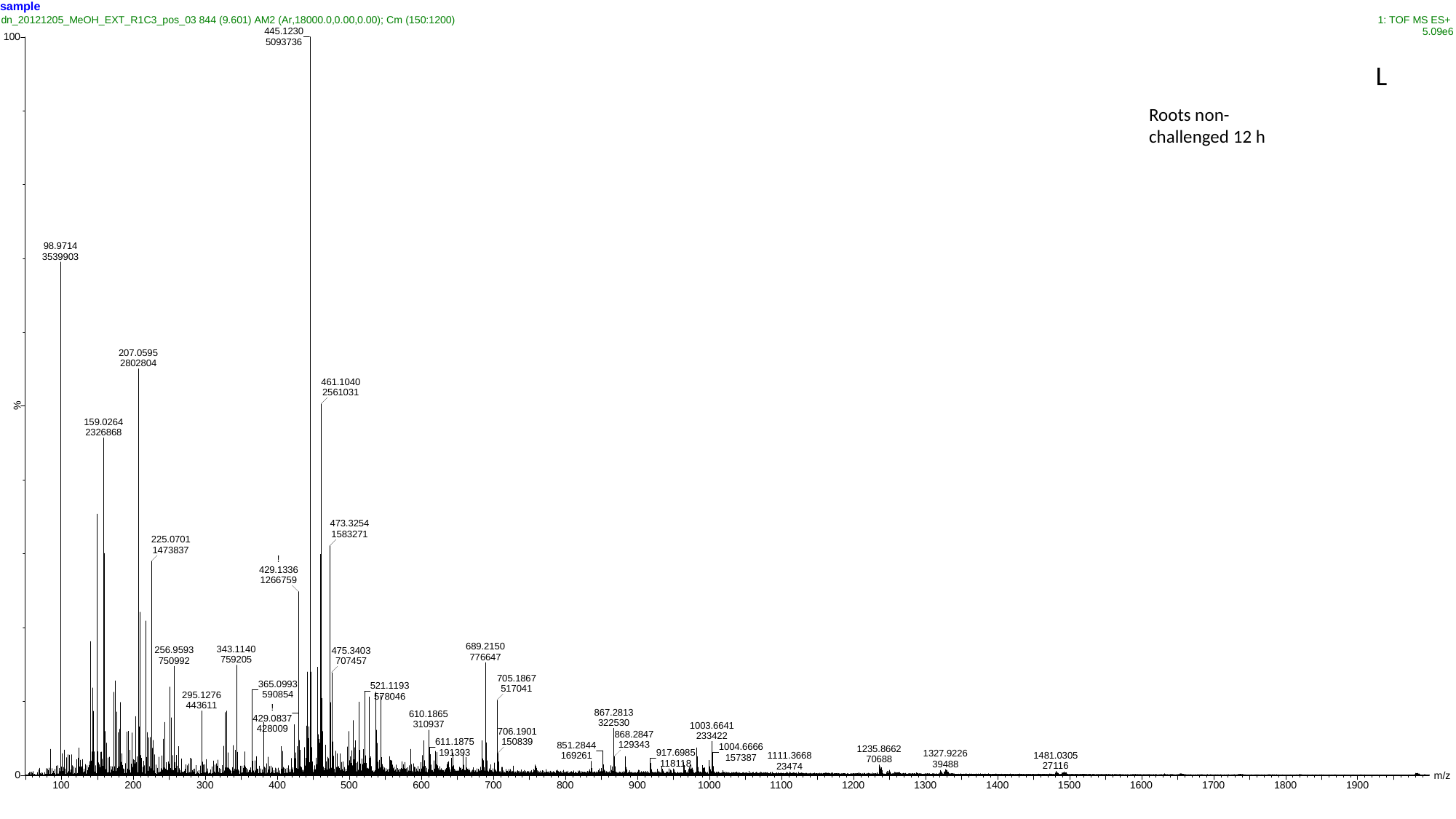

L
Roots non-challenged 12 h

## Slide 15
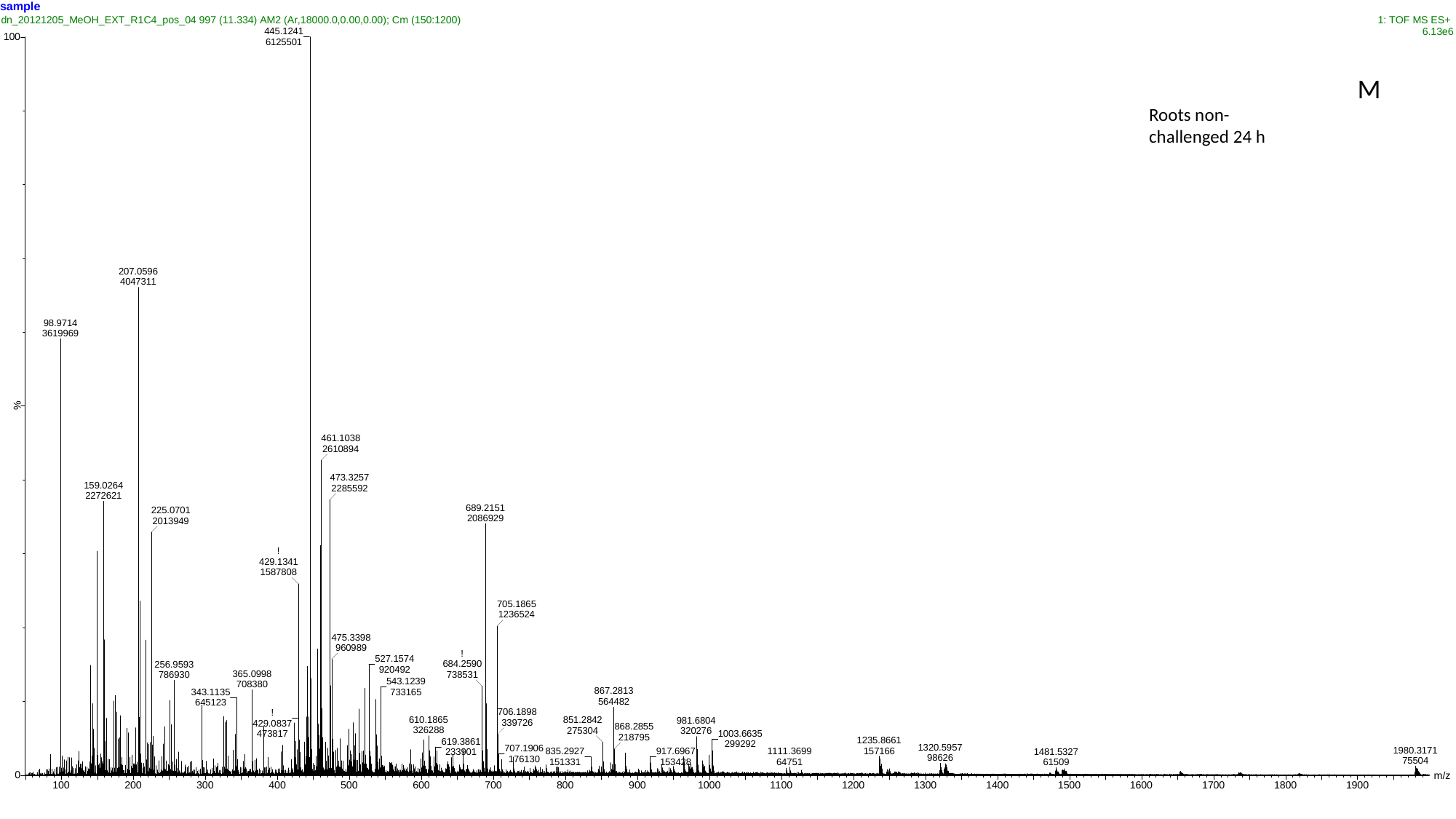

M
Roots non-challenged 24 h

## Slide 16
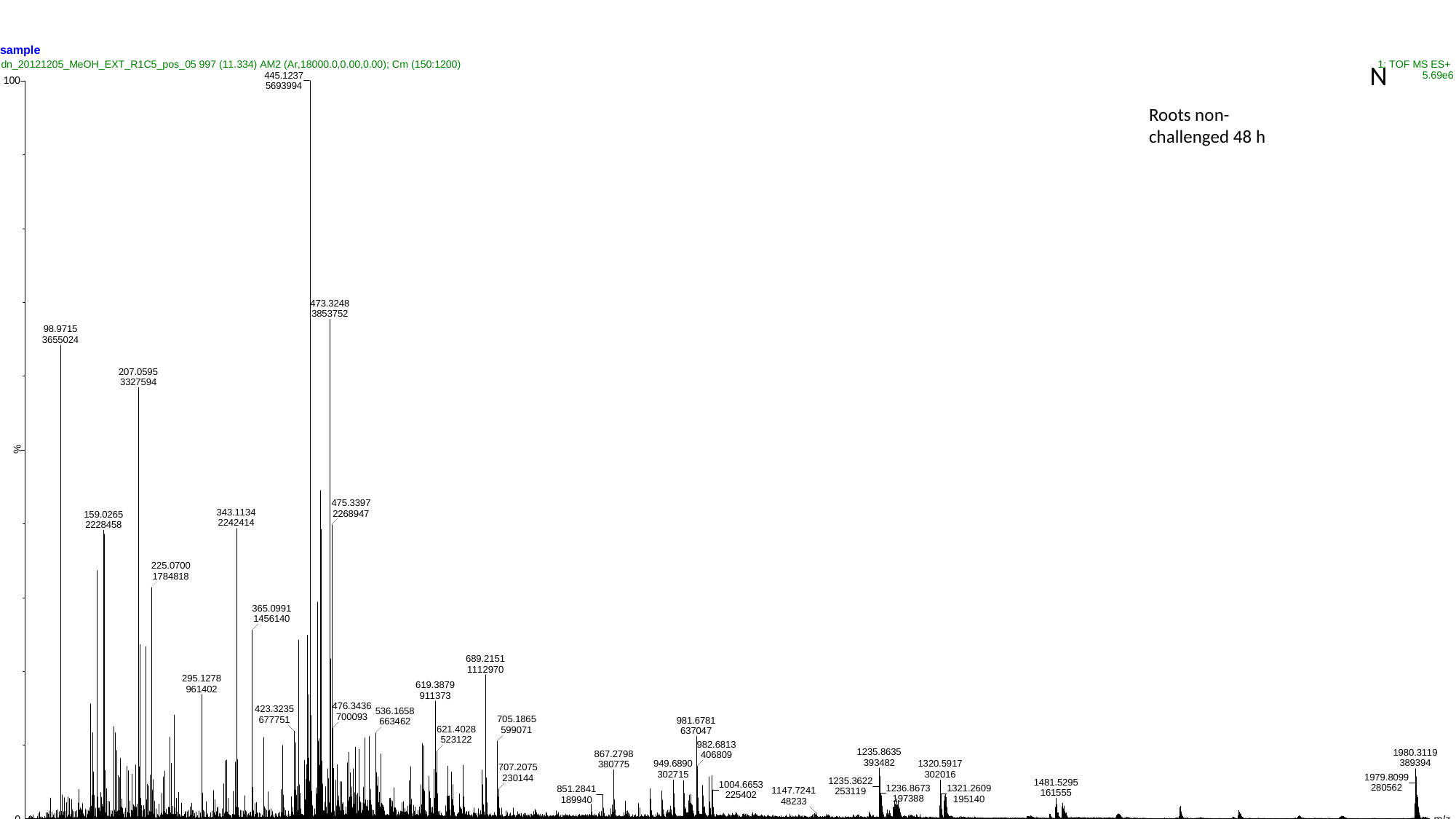

N
Roots non-challenged 48 h

## Slide 17
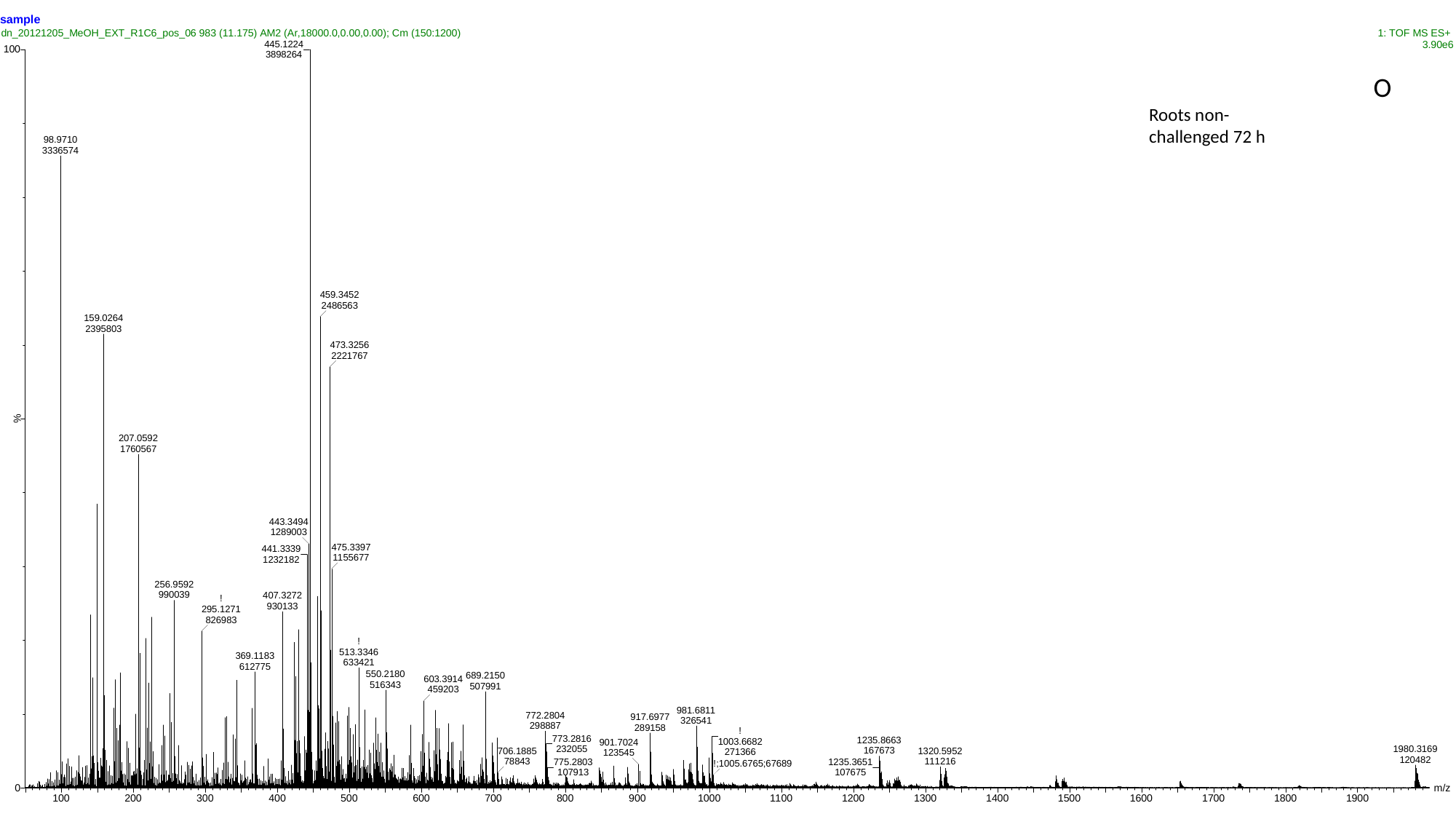

O
Roots non-challenged 72 h

## Slide 18
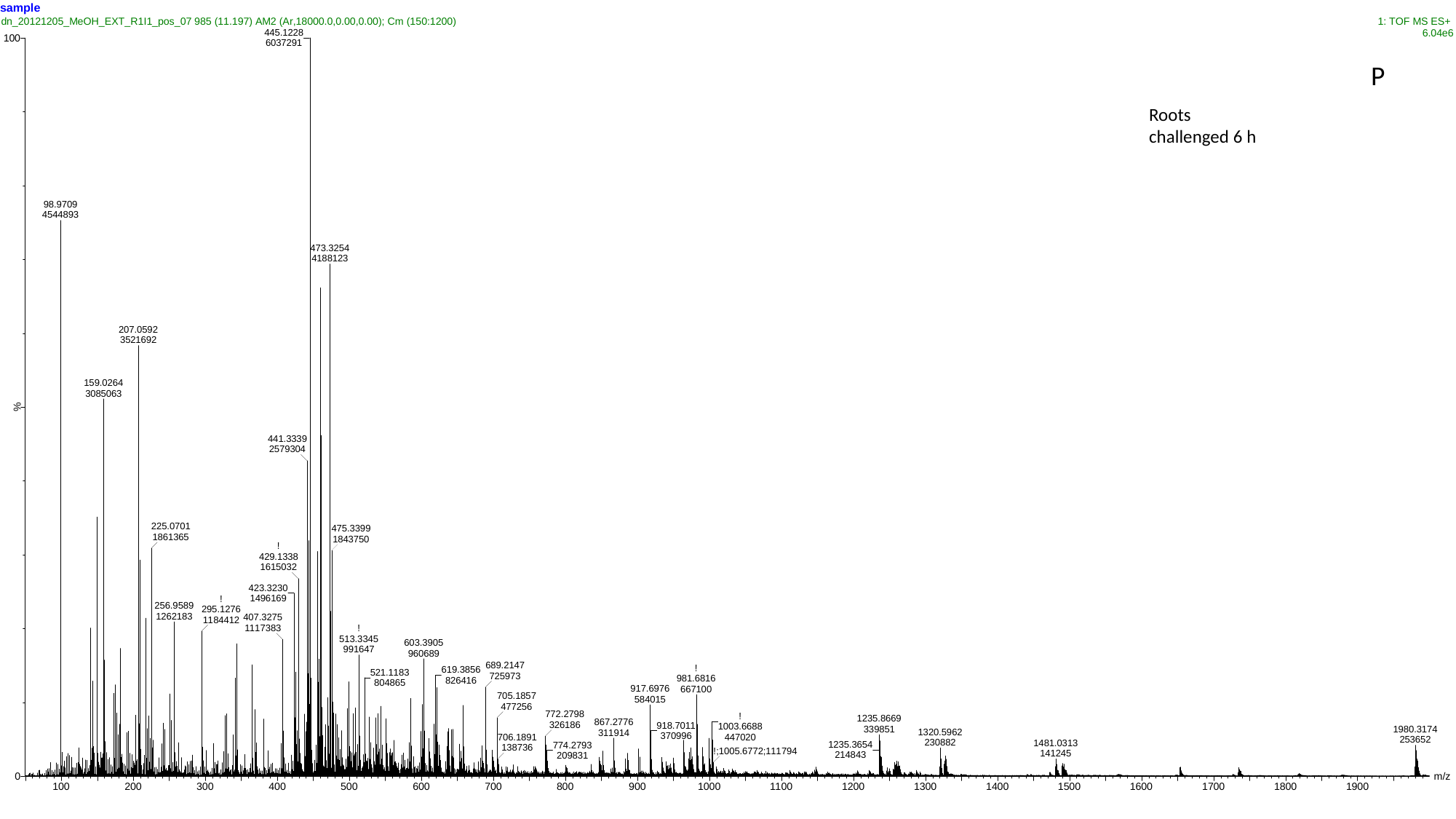

P
Roots challenged 6 h

## Slide 19
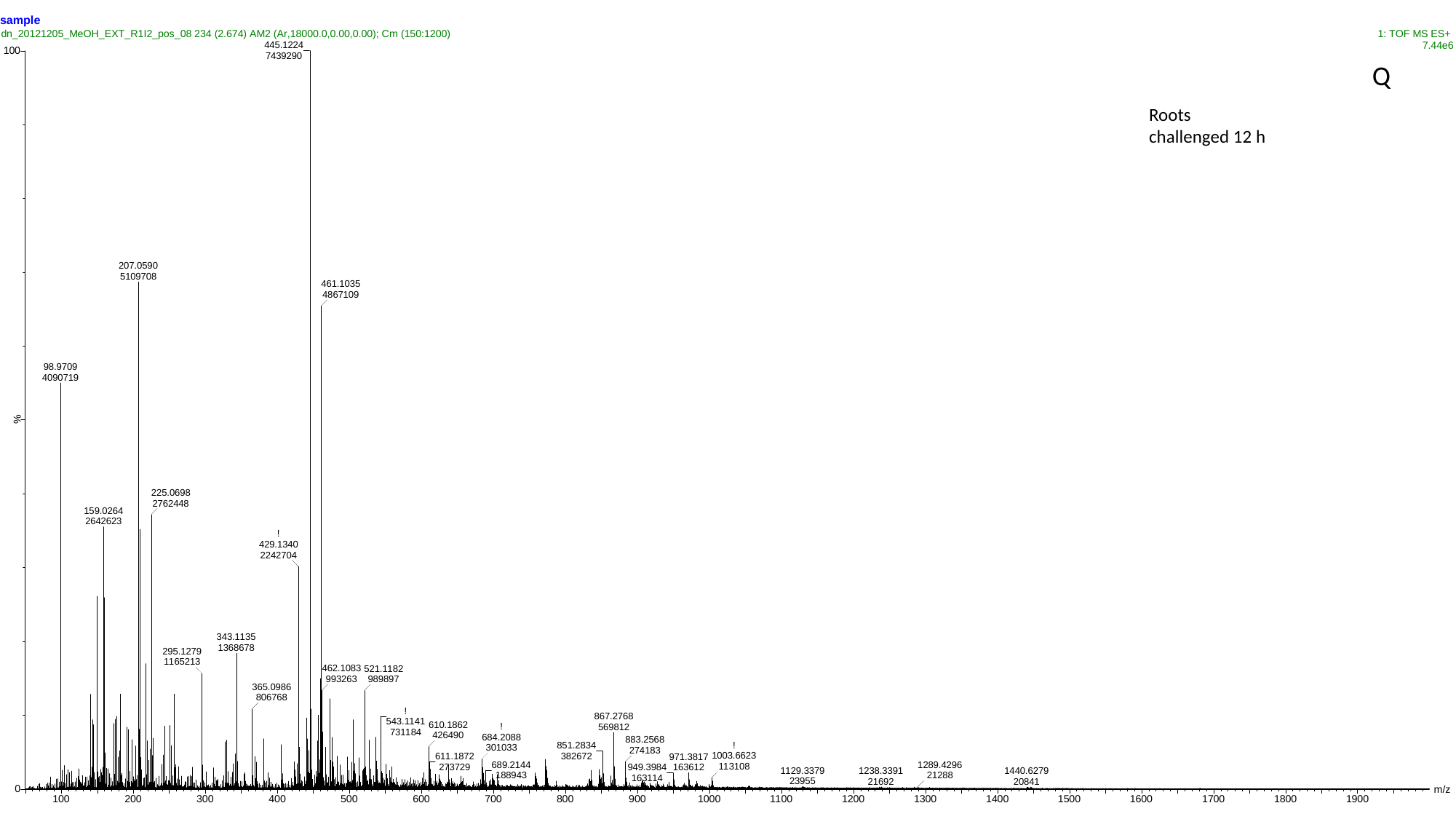

Q
Roots challenged 12 h

## Slide 20
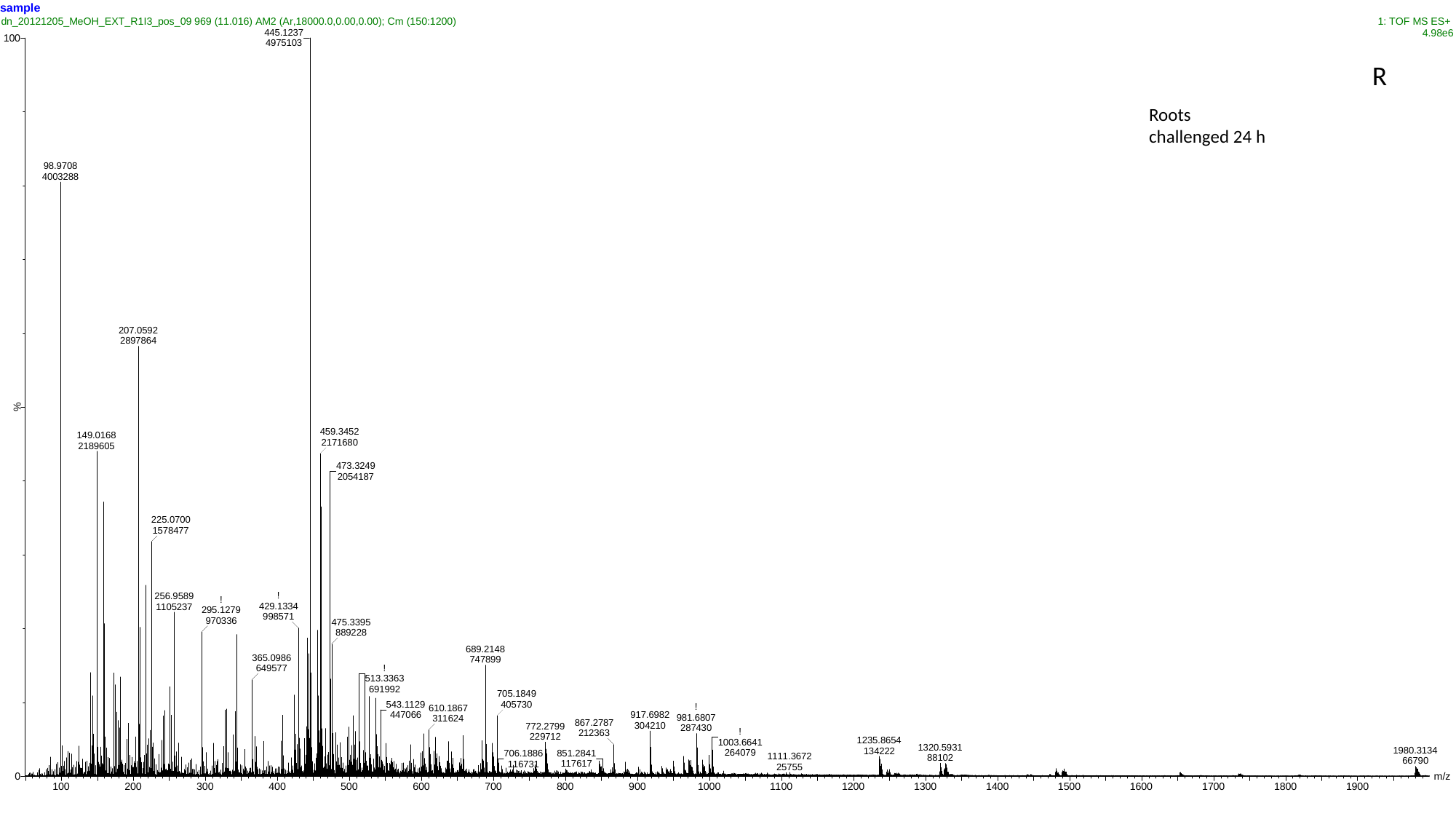

R
Roots challenged 24 h

## Slide 21
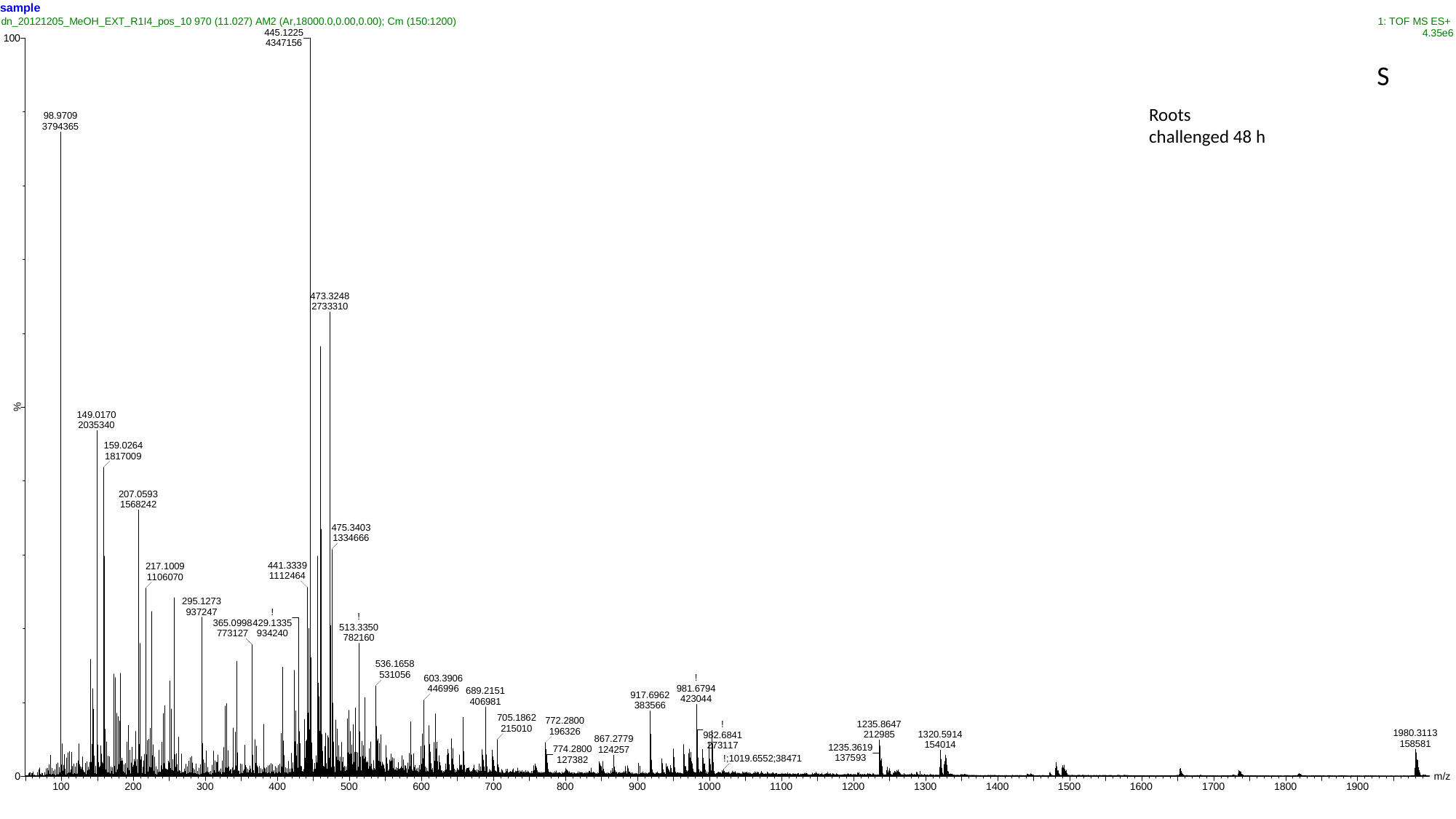

S
Roots challenged 48 h

## Slide 22
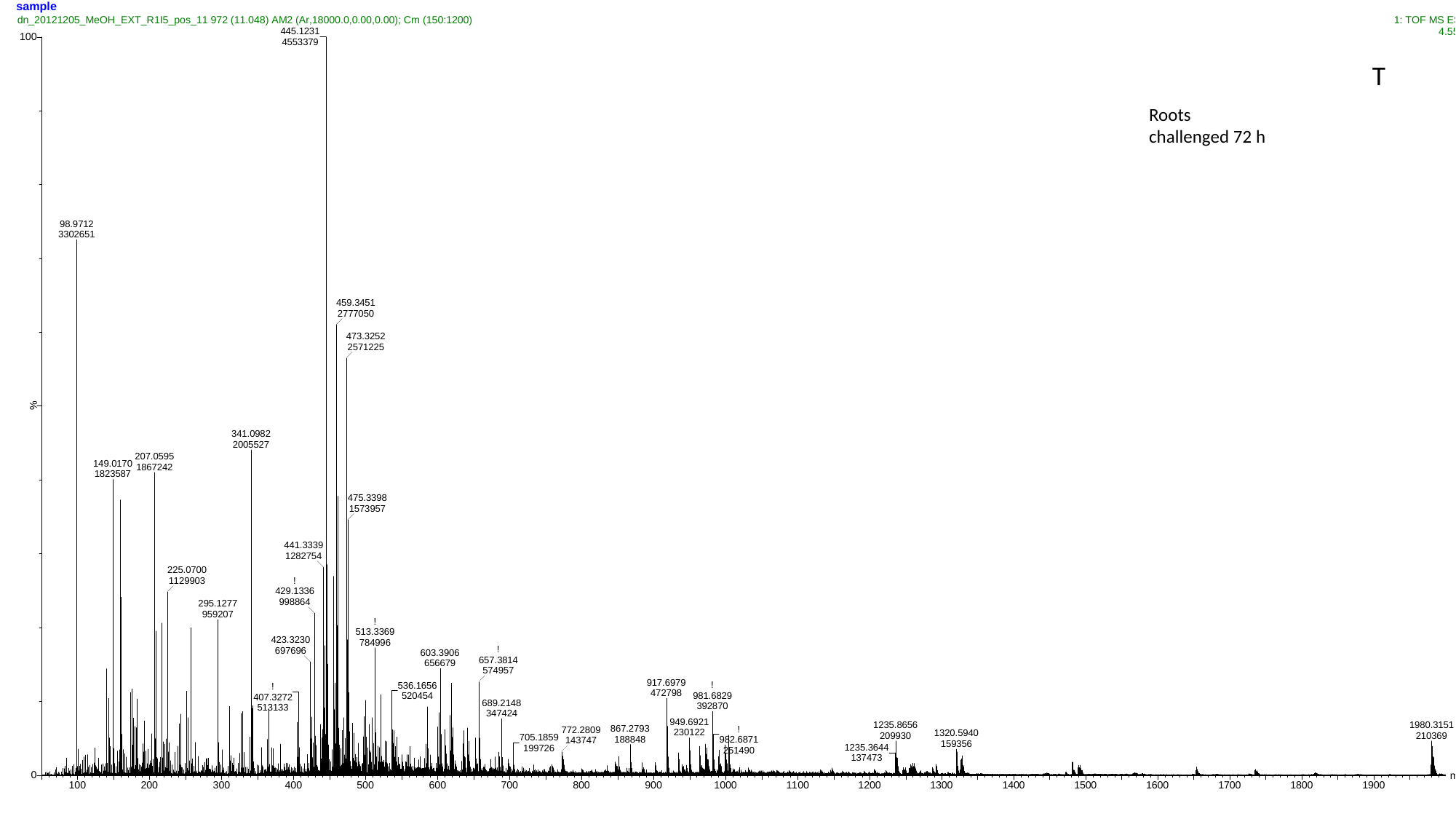

T
Roots challenged 72 h

## Slide 23
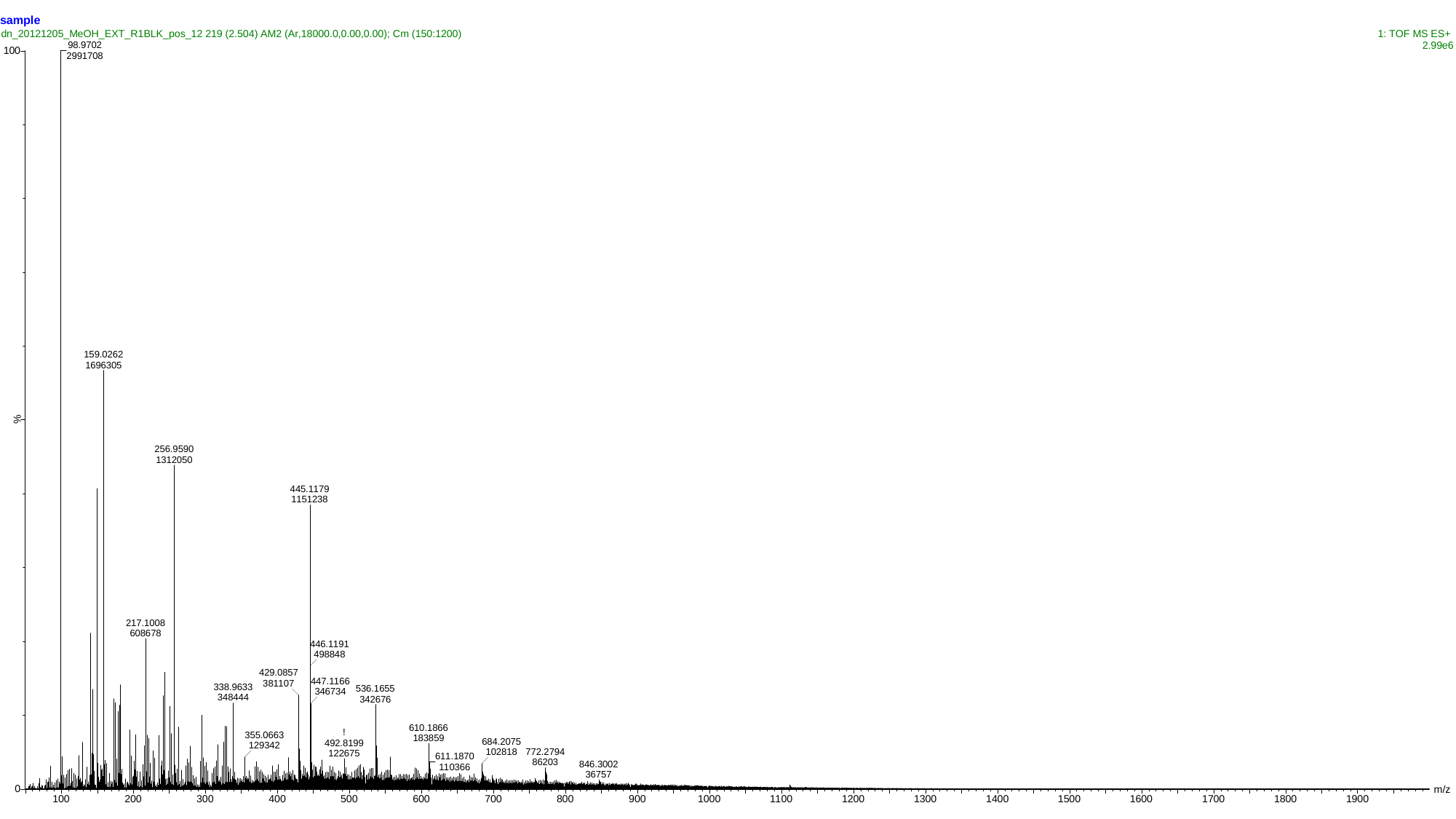

Supplement: Supplementary file 1 [file plants-12-01929-s001.zip › Fig. S1.pptx]
